# Theoretical and experimental assessment of genome-based prediction in landraces of allogamous crops

**DOI:** 10.1073/pnas.2121797119

**Published:** 2022-04-29

**Authors:** Armin C. Hölker, Manfred Mayer, Thomas Presterl, Eva Bauer, Milena Ouzunova, Albrecht E. Melchinger, Chris-Carolin Schön

**Affiliations:** ^a^Plant Breeding, TUM School of Life Sciences, Technical University of Munich, 85354 Freising, Germany;; ^b^KWS SAAT SE & Co. KGaA, 37574 Einbeck, Germany;; ^c^Campus Office, TUM School of Life Sciences, Technical University of Munich, 85354 Freising, Germany;; ^d^Institute of Plant Breeding, Seed Science and Population Genetics, University of Hohenheim, 70593 Stuttgart, Germany

**Keywords:** landraces, genomic selection, doubled haploids, gamete capture

## Abstract

Discovery and enrichment of favorable alleles in landraces are key to making them accessible for crop improvement. Here, we present two fundamentally different concepts for genome-based selection in landrace-derived maize populations, one based on doubled-haploid (DH) lines derived directly from individual landrace plants and the other based on crossing landrace plants to a capture line. For both types of populations, we show theoretically how allele frequencies of the ancestral landrace and the capture line translate into expectations for molecular and genetic variances. We show that the DH approach has clear advantages over gamete capture with generally higher prediction accuracies and no risk of masking valuable variation of the landrace. Prediction accuracies as high as 0.58 for dry matter yield in the DH population indicate high potential of genome-based selection. Based on a comparison among traits, we show that the genetic makeup of the capture line has great influence on the success of genome-based selection and that confounding effects between the alleles of the landrace and the capture line are best controlled for traits for which the capture line does not outperform the ancestral population per se or in testcrosses. Our results will guide the optimization of genome-enabled prebreeding schemes.

## Significance

Genetic variation inherent in landraces is essential for broadening the genetic diversity of our crops. This study pioneers the development of a theoretical framework to link molecular inventories of plant genetic resources to phenotypic variation, allowing an informed choice of landraces and their crossing partners. We show that genome-based prediction of genetic values can be implemented successfully in landrace-derived material, despite a strongly reduced level of relatedness compared with elite germplasm. Theoretical derivations are validated with unique experimental data collected on two different landraces. Our results are a pivotal contribution toward the optimization of genome-enabled prebreeding schemes.

Genetic improvement is essential to secure sustainable crop production. Future crops will have to combine high yield potential with major sustainability factors, such as stress tolerance and resource efficiency. To meet these demands, plant breeding will require a reservoir of genetic variation much larger than what is currently found in commercial varieties (1). For maize, it has been estimated that US breeding populations represent only 2% of the entire maize germplasm (2). In contrast, seed banks around the world harbor thousands of untapped landrace accessions (1, 3, 4). Revisiting this vast diversity of landraces is considered promising for elite germplasm improvement (1, 5–9), and developments in molecular, computational, and quantitative genetics open new avenues to make native diversity accessible.

Landraces have been shown to harbor beneficial alleles for traits with limited genetic variation in breeding populations (10), but for most agronomically important traits, they exhibit a substantial performance gap compared with elite germplasm (11–13). While for qualitative traits targeted introgression of favorable alleles discovered in landraces is possible, many traits have a polygenic foundation, which is determined by a large number of genes with small effects. Consequently, marker-based introgression of individual alleles is limited for those traits. Extracting inbred lines directly from landraces and selecting them for superior performance can close the performance gap only partially. Therefore, recurrent population improvement with additional rounds of recombination and selection is necessary to increase the frequency of favorable alleles before introducing landrace-derived genetic material into elite populations. Genome-based selection can accelerate this process, but the theoretical basis of its implementation in prebreeding still needs to be developed.

In outcrossing species, population improvement generally includes three distinct phases (14): 1) sampling candidates from the population to establish progeny for evaluation, 2) evaluating them in multienvironment field trials, and 3) recombining the best candidates to form the next cycle. In genome-based recurrent selection, genomic data are collected in the first phase, and together with data from the second phase, a statistical model is trained for prediction of breeding values of untested candidates from the same or future breeding cycles. The success of this approach depends strongly on the prediction accuracy that can be achieved with the training data. One major determinant is the type of progenies that can be derived from the ancestral landrace (e.g., inbred lines, full- or half-sib families). Additional factors are the quality of phenotyping expressed as the heritability (*h*^2^) of the target traits, the sample size (*N*), and the number of markers (*M*).

**Fig. 1.**
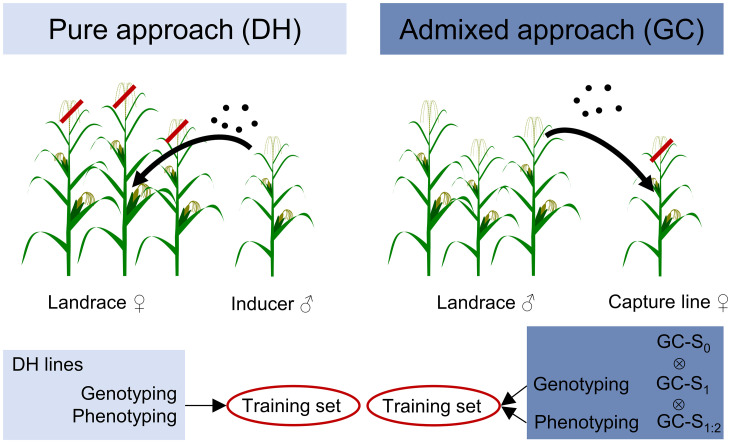
Scheme of population development for the pure and admixed approaches.

Here, we developed the quantitative genetic framework for two fundamentally different concepts for establishing training populations from landraces. The two concepts are displayed in Fig. 1 and differ with respect to the proportion of landrace genome and technical steps for their production. The “pure” approach entails the production of fully homozygous doubled-haploid (DH) lines from the ancestral landraces. The DH lines exhibit twice the additive genetic variance of the ancestral landrace and allow high-precision phenotyping. The “admixed” approach captures gametes of the landraces in a cross with an inbred (capture) line of different genetic background followed by subsequent selfing of the offspring. When the aim of the prebreeding program is the immediate development of superior inbred lines, a natural choice for the capture line would be a high-performing elite line to increase the usefulness of the resulting population compared with the pure approach. However, the use of an elite capture line has been shown to carry a high risk of reconstructing the elite genome, associated with a loss of landrace alleles in later selection steps (15). We, therefore, investigated the role of the capture line for the genetic improvement of landrace-derived populations with a focus on genome-based recurrent selection. We link generic theory with population-specific molecular parameters and experimental results on several traits, including yield, in four unique populations representing the pure and the admixed approach as well as two ancestral landraces.

## Results

### Molecular Variances of DH and Gamete Capture Populations Can Be Predicted.

**Fig. 2.**
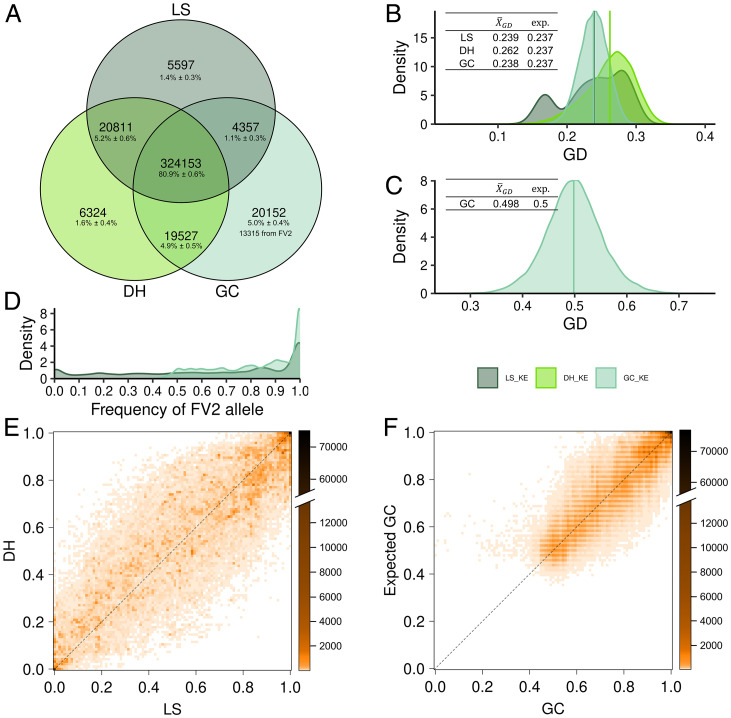
Venn diagram of the number and percentage of marker polymorphisms shared by and exclusive to the sample of the ancestral landrace (LS), DH lines, and GC lines of KE (*A*). Means and estimated densities of genetic distances (GD) between genotypes within LS, DH, and GC using all markers (*B*) and between GC lines and FV2 using only markers for which DH and LS were monomorphic for the allele not carried by FV2 (*C*). Estimated density of the frequency of the FV2 allele in LS and GC (*D*). Allele frequencies in DH vs. LS (*E*) and expected frequencies in GC (calculated from LS and known FV2 genotype) vs. observed GC (*F*). The calculated numbers of marker polymorphisms (*A*) are the result of sampling 80 gametes per population with 500 replications and are shown as the absolute number and percentage of polymorphic markers (± SD). In GC, the number of polymorphic markers resulting from the cross with FV2 (LS and DH monomorphic for the allele not carried by FV2) is shown as the average across 500 sampling replications. The tables in *B* and *C* show the means of the genetic distances and their expected values (calculated from LS allele frequencies). *B–F* are based on the whole set of lines (i.e., *N* = 48 [LS], *N* = 471 [DH], and *N* = 274 [GC]).

We developed populations of DH and gamete capture (GC) lines from two flint maize landraces, Kemater Landmais Gelb (KE) and Petkuser Ferdinand Rot (PE). The French inbred FV2 derived from population Lacaune served as the capture line. Both populations were produced from the same seed batch of the respective landrace, which we defined as the ancestral landrace. In addition to the derived DH and GC populations, a random sample from the ancestral landrace (LS) was genotyped. Across populations, 85 and 92% of the total 472,169 single nucleotide polymorphisms (SNPs) were polymorphic in KE and PE, respectively. The majority of the polymorphic markers (80.9% for KE and 78.4% for PE) segregated in all three populations (Fig. 2*A* and *SI Appendix*, Fig. S1*A*). In both landraces, each population showed a small percentage of segregating markers that were fixed in one or both of the other two populations due to independent sampling from the ancestral landrace. The capture line FV2 carried a SNP allele not present in the LS and DH lines at 13,315 (KE) and 11,488 (PE) genomic positions, thus contributing about half of the private polymorphisms of the GC lines. For both landraces, allele frequencies observed in DH and GC corresponded with allele frequencies estimated from LS and FV2 (Fig. 2 *E* and *F* and *SI Appendix*, Fig. S1 *E* and *F*).

Mean pairwise genetic distances of genotypes $({\bar{X}}_{GD}$) in the three types of populations are depicted in Fig. 2*B* and *SI Appendix*, Fig. S1*B* for landrace KE and PE, respectively. Under the assumption of Hardy–Weinberg equilibrium in the ancestral landrace and no selection, the expected ${\bar{X}}_{GD}$ in LS and DH is a function of the ancestral allele frequencies with ${\bar{X}}_{GD(LS)}=\;{\bar{X}}_{GD(DH)}$ (*SI Appendix*, *SI* *Text A1* and Table A1). In the GC populations, the allele frequencies of the capture line need to be accounted for. In the experimental LS and DH populations, mean and range of pairwise genetic distances were similar but not identical, with ${\bar{X}}_{GD(LS)}<\;{\bar{X}}_{GD(DH)}$ in KE and vice versa in PE. The more pronounced difference between LS and DH in KE was most likely the result of mild population admixture in the LS, which is reflected by an excess of closely related genotypes (Fig. 2*B*). Mean genetic distances between GC lines and the capture line FV2 calculated based on SNPs for which the LS and DH were monomorphic for the allele not carried by FV2 were in agreement with the expected value 0.5 in both landraces (Fig. 2*C* and *SI Appendix*, Fig. S1*C*). For this reduced set of markers, the variation of genetic distances to FV2 in GC reflects the effect of Mendelian sampling, as GC-S_0_ plants are fully heterozygous, and the resulting genetic distances should be equivalent to what is expected in the F_2_ generation of a biparental cross.

**Table 1.** Quantitative-genetic expectations of means and genetic variances for per se (PP) and testcross (TP) performance in the sample of the ancestral landrace (LS), derived DH, and GC lines

Population	Coefficient of parameters^†^									
	Population mean^‡^ $\bar{x}$				Genetic variances					
					Primary variance		Variance within families		Total variance	
	a+Δ	(p−(1−p))a	[d]= $2p(1-p)d_{12}$	$\left[d^{*}\right]=$ $pd_{1x}+(1-p)d_{2x}$	$\sigma_{A}^{2}$	$\sigma_{A^{*}}^{2}$	$\sigma_{A}^{2}$	$\sigma_{A^{*}}^{2}$	$\sigma_{A}^{2}$	$\sigma_{A^{*}}^{2}$
LS	0	1	1	0	1	0	—	—	1	0
DH	0	1	0	0	2	0	0	0	2	0
GC-S_1:2_	1/2	1/2	0	1/4	3/4	1/4	1/8	1/8	7/8	3/8
GC-S_1:∞_	1/2	1/2	0	0	3/4	1/4	1/4	1/4	1	1/2
FV2	1	0	0	0	—	—	—	—	—	—

For GC lines, the total genetic variance is decomposed into the primary variance between families as observed for GC-S_1:2_ lines in this study and the variance within families.

^†^Parameters [d] and $\left[d^{*}\right]$ are not required for TP.

^‡^p and (1−p) refer to the frequencies of alleles $A_{1}$ and $A_{2}$ in LS, respectively. a and a+Δ refer to the additive effects in LS and the capture line, respectively, with different meanings for PP and TP. [d] and $\left[d^{*}\right]$ refer to the contribution of dominance effects to the PP of LS and GC-S_1:2_, respectively, where $d_{12}$, $d_{1x}$, and $d_{2x}$ refer to the dominance effect of genotypes $A_{1}A_{2}$, $A_{1}A_{x}$, and $A_{2}A_{x}$, respectively, with $A_{x}$ being the allele of the capture line. $\sigma_{A}^{2}$ refers to the additive variance inherent in the ancestral landrace, with $\sigma_{A}^{2}=2p(1-p)a^{2}$. $\sigma_{A^{*}}^{2}$ refers to the additive variance resulting from the effects of the capture line alleles, with $\sigma_{A^{*}}^{2}=2{\left(\left(1-p\right)a+\text{Δ}/2\right)}^{2}$ (details are in *SI Appendix*, *SI* *Text A2*).

For all three types of populations, we derived expectations of the total molecular variance and its decomposition between and within genotypes assuming absence of selection. For a single locus, the total molecular variance calculated based on biallelic SNP allele frequencies is expected to be identical for DH and the sample from the ancestral landrace (LS) with $\varsigma_{DH}^{2}=\varsigma_{LS}^{2}=2p\left(1-p\right)$, with *p* being the frequency of the allele carried by the capture line (*SI Appendix*, Table A3). For GC, the expected total molecular variance amounts to $\varsigma_{GC-S_{1}}^{2}=0.5\left(1+p\right)\left(1-p\right)$ (*SI Appendix*, Table A3). Consequently, for a given locus, $\varsigma_{GC-S_{1}}^{2}\geq \varsigma_{LS}^{2}$ if and only if the allele present in the capture line has frequency p ≤1/3 in the ancestral landrace. Loci where the ancestral landrace and therefore also its sample are fixed for an allele different from FV2 (i.e., p=0) contribute maximally to $\varsigma_{GC-S_{1}}^{2}$ but not to $\varsigma_{LS}^{2}$ and $\varsigma_{DH}^{2}$. Our theoretical results demonstrate the importance of the genetic makeup of the capture line for building the GC. Here, the capture line FV2 contributed new alleles (p=0 in LS and DH) at 2% (PE) and 3% (KE) of all polymorphic SNP positions, and the proportion of SNPs with p ≤1/3 in LS was about 25% in both landraces (Fig. 2*D* and *SI Appendix*, Fig. S1*D*). Thus, the observed molecular variances for LS, DH, and GC (*SI Appendix*, Table S1) meet expectations.

**Fig. 3.**
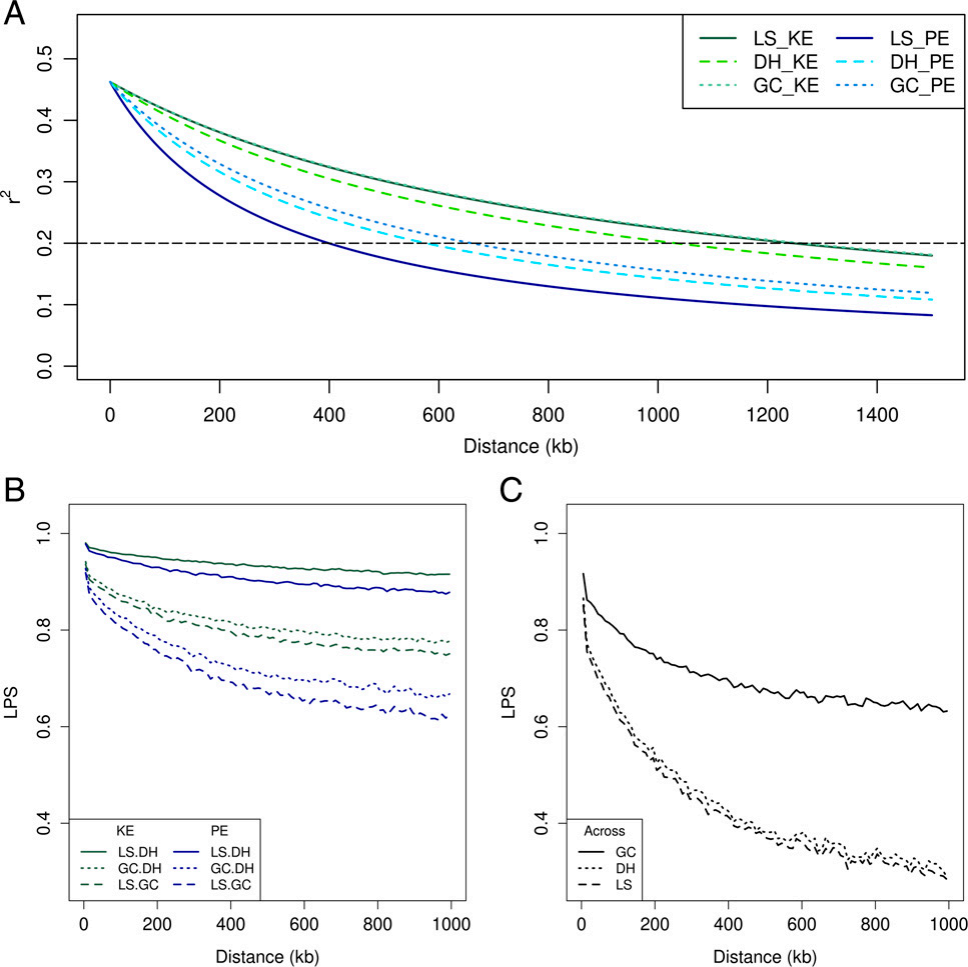
Decay of LD with physical distance for the sample of the ancestral landrace (LS), the DH lines, and the GC lines of landraces KE and PE (*A*). Linkage phase similarities (LPS) for pairwise comparisons of the three types of populations within each landrace (*B*) and LPS for pairwise comparisons of the same type of population across the two landraces (*C*). For all calculations, 94 gametes were randomly sampled for each group.

The linkage disequilibrium (LD) decay distance (*δ*), for which the pairwise LD of markers on the same chromosome was greater than *r*^2^ > 0.2, was slightly higher in the GC than in the DH lines and was higher in populations derived from KE (1,032 ≤ *δ* ≤ 1,263 kb) than from PE (399 ≤ *δ* ≤ 660 kb) (Fig. 3*A*). Within landraces, linkage-phase similarities (LPS) were high for the pairwise comparison of LS and DH but substantially reduced for LS and GC (Fig. 3*B*). Across landraces, LPS for the pairwise comparison of the same type of population was low for LS and DH but moderate to high for GC (Fig. 3*C*). Average LD between markers on different chromosomes was negligible in all populations and both landraces.

### Experimental and Theoretical Results Are in Good Agreement for All Populations.

The conceptual differences between the pure and the admixed approach with respect to means and genetic variances in DH and GC are visualized in *SI Appendix*, Fig. A1 based on the theoretical expectations given in Table 1 and *SI Appendix*, Table A2. In hybrid breeding, selection candidates are evaluated not only for their per se performance (PP) but mainly, for their combining ability with a tester from a different heterotic group. We, therefore, considered both the PP of the LS and of GC-S_1:2_ and DH lines as well as their testcross performance (TP) with an inbred line from the dent heterotic pool. Assuming absence of epistasis, the PP of fully inbred generations (DH lines, GC-S_1:∞_ lines) and of all testcrosses can be described with a purely additive model (Table 1 and *SI Appendix*, *SI* *Text A2* and Table A2). For PP of the LS and for GC-S_1:2_ lines, the mean and dispersion of the genotypic values depend on unknown landrace- and capture line specific dominance effects [d] and $\left[d^{*}\right]$, respectively (Table 1).

**Fig. 4.**
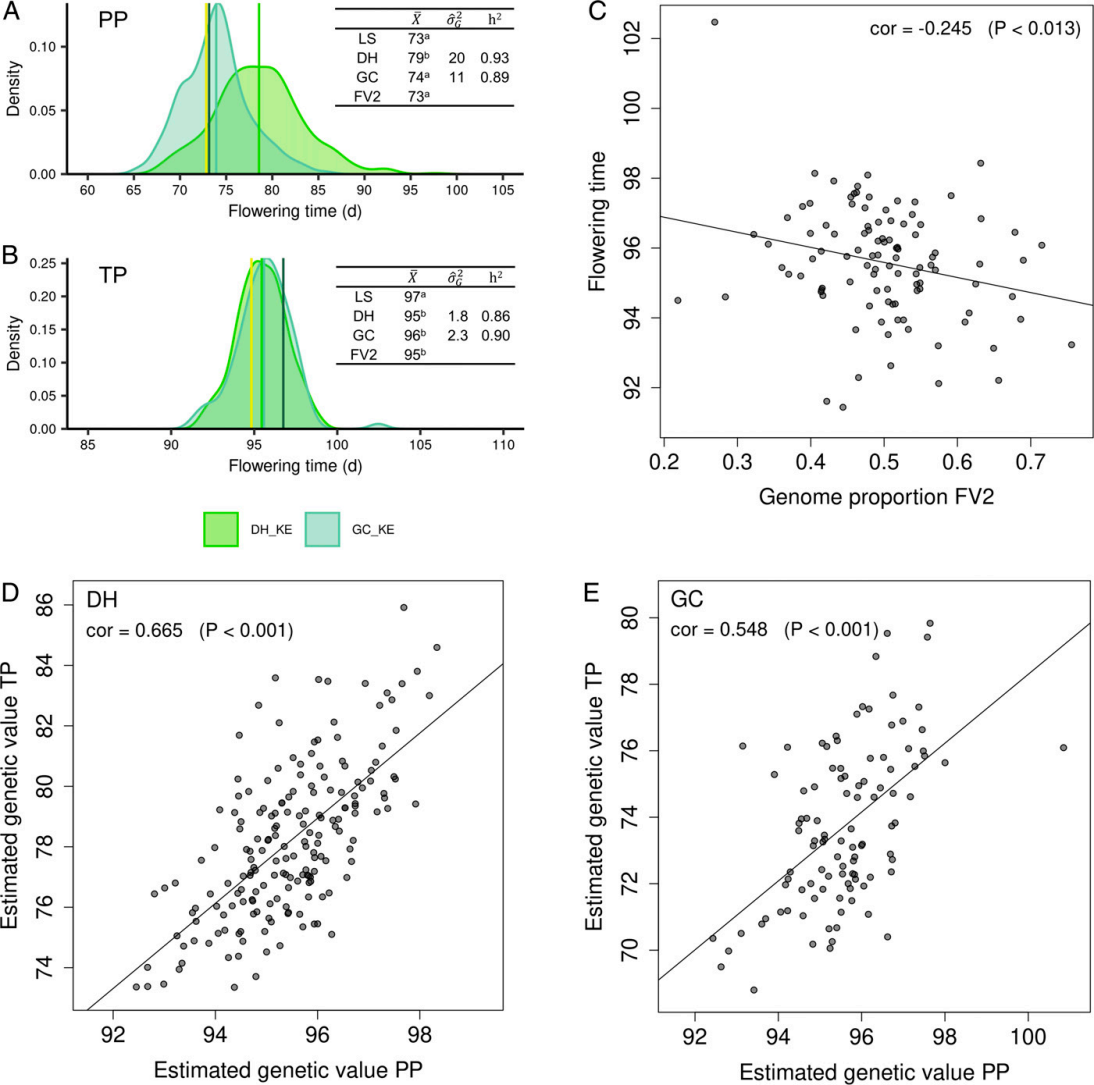
Estimated densities showing the distribution of phenotypic values for per se performance (PP; *A*) and testcross performance (TP; *B*) of the DH and GC lines for landrace KE, scatterplots of proportions of FV2 genome vs. TP for flowering time (*C*), and estimated genetic values of PP vs. estimated genetic values of TP for flowering time in DH (*D*) and GC (*E*) lines. In *A* and *B*, the means (vertical lines) of the landrace sample (LS, dark green) and the capture line FV2 (yellow) are indicated, and the tables show the means ($\bar{X}$), genetic variances (${\hat{\sigma }}_{g}^{2}$), and heritabilities (*h*^2^). Means with a shared letter are not significantly different (*P* > 0.05). *C*–*E* indicate the Pearson correlation coefficients and corresponding *P* values of the shown correlations.

In the following, we use the trait flowering time exemplarily to link theoretical and experimental results (Fig. 4 and *SI Appendix*, Fig. S2). Phenotypic values should be indicative of genotypic values, as heritabilities were high, ranging between 0.85 and 0.93 (Fig. 4 *A* and *B* and *SI Appendix*, Fig. S2 *A* and *B*). The LS flowered significantly earlier than most DH lines, and estimates of [d] amounted to about 7% of the LS performance for both landraces. The inbred capture line FV2 flowered significantly earlier than the mean of the DH lines from KE and PE, pointing to an enrichment of early flowering alleles in FV2. Mean flowering time of GC lines was significantly earlier than of DH lines. Under an additive model, the mean of the GC lines is expected to lie exactly between the mean of the DH lines and the capture line but was shifted toward FV2 in both landraces, indicating capture line specific dominance effects $\left[d^{*}\right]$ contributing to GC per se performance. In the testcrosses, differences between the capture line and the mean of the DH lines were attenuated, and consequently, mean DH and GC testcross performance was not significantly different in both landraces.

We derived expected genetic variances of the DH and GC populations (Table 1). The DH delivers the maximum additive genetic variance inherent in the respective landrace with $\sigma_{g(DH)}^{2}=\;2\sigma_{A}^{2}$. The additive genetic variance among GC lines is $\sigma_{g(GC-S_{1:2})}^{2}=\;\frac{3}{4}\sigma_{A}^{2}+\frac{1}{4}\sigma_{A*}^{2}$ (Table 1), with $\sigma_{A*}^{2}=2{\left(\left(1-p\right)a+\text{Δ}/2\right)}^{2}$ being a function of the effect and frequency of the alleles in the ancestral landrace and the effects of alleles originating from the capture line. If for a given trait the capture line contributes only alleles present in the ancestral landrace, as would be the case for a random DH line derived from the ancestral landrace, the genetic variance of the GC lines should be half the genetic variance among DH lines as $\sigma_{A*}^{2}$ = $\;\sigma_{A}^{2}$ (*SI Appendix*, *SI* *Text A2*). If the capture line contributes alleles not present in the landrace at many loci and the allelic effects at these loci differ substantially from the respective landrace alleles, the genetic variance among GC lines can be equal to or even larger than the variance among DH lines.

Estimates of genetic variances for flowering time were significant for PP and TP in both landraces (Fig. 4 *A* and *B* and *SI Appendix*, Fig. S2 *A* and *B*). For PP, the ratio $\sigma_{g\left(GC\right)}^{2}/\sigma_{g(DH)}^{2}\;$ was 0.55 in KE and 0.67 in PE. The results suggest that the additive genetic variance $\sigma_{A*}^{2}$ generated by crossing the landrace with FV2 did not differ substantially from $\sigma_{A}^{2}$, despite the enrichment of earliness alleles in FV2. In the testcrosses, however, the ratio $\sigma_{g\left(GC\right)}^{2}/\sigma_{g(DH)}^{2}\;$ was 1.28 in KE and 1.35 in PE, indicating $\sigma_{A*}^{2}>\sigma_{A}^{2}$. The reduction in $\sigma_{g}^{2}$ in the testcrosses compared with per se performance was much higher for DH than GC lines. These results indicate that dominance interactions with the tester allele differed for the DH and FV2, and consequently also for GC, in both landraces. Nevertheless, genetic covariances between PP and TP were significant for both types of populations and both landraces. Genotypic correlations as well as correlations of estimated genetic values between PP and TP for flowering time were higher for DH than for GC lines (Fig. 4 *D* and *E* and *SI Appendix*, Fig. S2 *D* and *E* and Table S2).

One concern with the GC population is the overrepresentation of the capture line genome in the progeny after selection. The proportion of FV2 genome of GC-S_1_ plants determined with the reduced marker set (SNP alleles with p=0 in LS and DH) ranged from 21.9 to 75.6% in KE and from 21.5 to 73.1% in PE, with averages of 50.3 and 50.2%, respectively, meeting expectations. A significant correlation of FV2 genome proportion and phenotypic performance was observed for flowering time only in the testcrosses of KE. As expected, the correlation was negative but weak (*r* = −0.25) (Fig. 4*C* and *SI Appendix*, Fig. S2*C*), demonstrating that GC lines enriched with earliness alleles can be selected without strong overrepresentation of the FV2 genome.

Results for the other traits are presented in *SI Appendix*, Figs. S3 and S4. In general, experimental results were in agreement with theoretical expectations and highly consistent across landraces. Estimates of [d] for the two plant height traits amounted on average to about 26% relative to the performance of the LS. The mean of the GC lines for plant height was about halfway between LS and FV2, also indicating dominant type of gene action. For all traits, the ratio of genetic variances $\sigma_{g\left(GC\right)}^{2}/\sigma_{g(DH)}^{2}\;$ followed the same trend as shown for flowering time in both landraces for PP and TP. Correlations of FV2 genome proportion and observed phenotypic performance were not significant for all traits and both landraces (except flowering time in testcrosses as described above). For early plant height, the GC lines showed only low (PE) or nonsignificant (KE) genetic correlations between PP and TP, while for DH lines, they were intermediate to high (*SI Appendix*, Table S2).

### Population Type Determines Accuracy of Genomic Prediction.

**Fig. 5.**
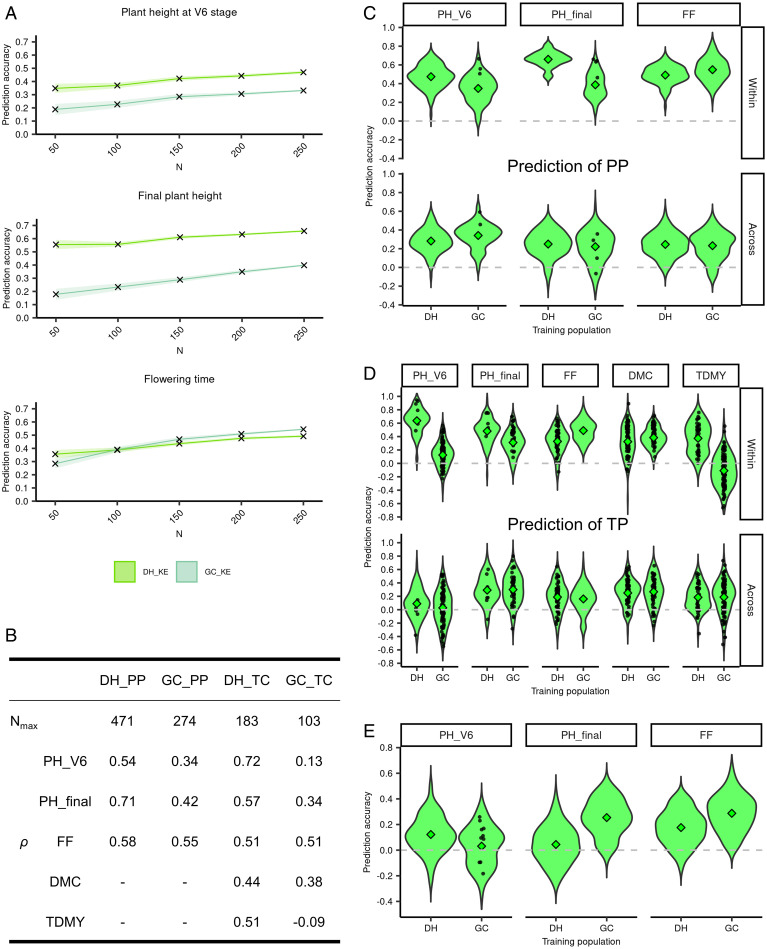
Prediction accuracy (*ρ*) in landrace KE for per se performance (PP) in the DH and GC lines as a function of sample size *N* (*A*), for prediction of PP and testcross performance (TP) at the maximum available number of lines (*N*_max_; *B*), for predictions within and across populations for PP (*C*) and TP (*D*) in DH and GC, and for across-landrace prediction for PP from KE (training on PE; *E*). Traits are plant height at V6 stage (PH_V6), final plant height (PH_final), and flowering time (FF) in PP and TP and dry matter content (DMC) and total dry matter yield (TDMY) in TP. For each *N* (*A*), sampling of lines was repeated 100 times, and 10 times fivefold cross-validation was carried out within each sample, yielding the basis for calculating the presented means and 95% quantiles (shaded areas around the curve). Prediction across and within populations as well as across landraces was carried out by randomly sampling *N* = 200 and *N* = 75 lines for training in PP (*C* and *E*) and TP (*D*), respectively, for predicting *N* = 50 (PP; *C* and *E*) or *N* = 25 (*D*) genotypes of the same or corresponding population (*C* and *D*) or the same population of the other landrace (*E*). Sampling was repeated 100 times. The violin plots (*C*–*E*) show all 100 values, with the diamonds indicating the means. Black dots show values of the prediction accuracy estimated from models where the genomic variance estimate was not significant (likelihood-ratio-test, *P* > 0.05).

The accuracy *ρ* of genome-based prediction is the success criterion for genomic selection. Increasing the sample size of the training set affected the magnitude and precision of *ρ*, and no plateau was reached up to *N* = 250 (Fig. 5*A* and *SI Appendix*, Fig. S5*A*). With respect to marker density, an increase in prediction accuracy could be observed up to 15,000 SNPs (*SI Appendix*, Fig. S6). Prediction accuracies *ρ* were consistently higher in DH lines compared with GC lines for the two plant height traits for all tested sample sizes of the training set. Differences were most pronounced for small sample sizes. For flowering time, differences between the two types of populations were negligible. Yield and dry matter content were assessed in testcrosses only. Accuracies for yield exceeded 0.5 in DH lines even with sample sizes *N* < 200 (Fig. 5*B* and *SI Appendix*, Fig. S5*B*). However, in the GC lines, prediction for yield failed (*ρ* = −0.09 in KE) and was very low for early plant height. The strong decrease in prediction accuracies of testcross traits in the GC can partially be accounted for by a combination of nonsignificant genetic variances in a high number of training sets (Fig. 5*D* and *SI Appendix*, Fig. S5*D*) and the limited size of the prediction sets in cross-validation (*N* = 25). In DH lines, however, testcross accuracies exceeded those of per se performance in some cases, despite lower genetic variances, lower heritabilities, and smaller training set size (plant height at V6 stage in KE, flowering time in PE).

Prediction accuracies across the two types of populations (Fig. 5 *C* and *D* and *SI Appendix*, Fig. S5 *C* and *D*) were low (0.20 to 0.49 for lines per se, 0.03 to 0.41 for testcrosses). The higher accuracies observed within DH lines (e.g., for final plant height) were not reflected in prediction across populations. Accuracies were similar irrespective if the prediction model was trained on DH to predict GC or vice versa. We also investigated if combining the two populations yielded a predictive advantage over within-population prediction. Despite a substantial increase in sample size, accuracies changed only marginally (from 0.53 to 0.55 on average across traits) (*SI Appendix*, Table S3), which was not expected considering the increase in prediction accuracy within populations with increasing *N* (Fig. 5*A* and *SI Appendix*, Fig. S5*A*).

Prediction across landraces (e.g., using DH lines of KE for training and DH lines of PE for prediction or vice versa) yielded estimates of *ρ* close to zero for DH lines irrespective of which landrace was used for model training (Fig. 5*E* and *SI Appendix*, Fig. S5*E*). For GC lines, higher values were obtained especially for final plant height and flowering time (0.25 ≤ *ρ* ≤ 0.29), most likely due to shared haplotypes originating from FV2, resulting in much higher linkage phase similarities of GC compared with DH populations (Fig. 3*C*).

Estimates of *ρ* varied substantially for the different (cross-)validation runs (Fig. 5 and *SI Appendix*, Fig. S5) within and across populations as well as across landraces. In testcrosses with a training set size of *N* = 75 (per se *N* = 200), this was most pronounced. The variation was in part attributable to nonsignificant estimates of the genetic variance in the training set, which was more common in GC and most pronounced in testcross prediction.

## Discussion

Extraction of beneficial haplotypes from landraces is a long-term endeavor. In landrace genomes, favorable alleles for one trait are often in high LD with unfavorable alleles for the same or other traits, and consequently several rounds of recombination and selection are required to close the performance gap to elite material and reduce linkage drag (16). With this study, we aimed to fill the knowledge gap on genome-based prediction accuracies that can be achieved in landrace-derived material in outcrossing species.

### Prediction Accuracies Are High in Landrace-Derived DH Populations.

Prediction accuracies achieved in this study with landrace-derived DH lines clearly demonstrate that genome-based selection has great potential. Cross-validated accuracies for prediction of total dry matter yield were as high as 0.58 in testcrosses of PE DH lines (0.51 in KE) and even higher for other traits, despite sample sizes of less than 150 DH lines in the training set. Correlated estimated genetic values for PP and TP indicate effective genomic selection for traits like flowering time and plant height on the per se level, carrying over to correlated response for TP.

In landrace-derived DH populations, prediction accuracies should be merely a function of LD between markers and quantitative trait loci (QTL), as gametes are sampled at random from the ancestral population. Thus, it was surprising that accuracies were of similar magnitude as reported for elite maize germplasm with much higher LD and substantial relatedness between genotypes (17). Inflation of accuracies caused by DH lines with extreme values due to strong inbreeding depression could be ruled out by investigating cross-validation prediction sets manually. Hidden relatedness and population structure in the DH population, both factors that might inflate prediction accuracy, were not observed when investigating pairwise genetic distances of DH lines (Fig. 2 and *SI Appendix*, Fig. S1).

We, therefore, conclude that in DH populations derived from landraces preselected for molecular and phenotypic properties as suggested by Mayer et al. (18), prediction accuracies of 0.5 or higher can be considered a realistic benchmark in genome-based selection of complex traits with training set sizes of *N* ≥ 200 due to large additive genetic variance, high heritabilities, and moderate LD.

### Efficiency of the Admixed Approach.

Some landrace populations carry high genetic load, leading to low efficiency of DH production. Thus, crossing the landrace with an inbred capture line from a different genetic background might be the only option to avoid homozygous deleterious allele combinations. So, what are the consequences for prediction accuracies in comparison with DH populations? As expected from theory and observed in the experimental populations of this study, average allele frequencies of polymorphic SNPs were shifted toward more unbalanced allele frequencies in the GC lines (Fig. 2 and *SI Appendix*, Fig. S1), affecting locus-specific contributions to the total genetic variance. If the capture line carries an allele present in the ancestral landrace, the locus-specific variance in the GC decreases compared with the DH population, except for loci with extreme allele frequencies in the ancestral landrace (p ≤1/6) (*SI Appendix*, *SI* *Text A3*). If the capture line carries an allele not present in the ancestral landrace, the locus-specific variance in the GC will depend on the effect of this allele as well as on the frequencies and effects of the alleles in the ancestral landrace. If the allele of the capture line exhibits dominance over the landrace alleles (i.e., $\left[d^{*}\right]$ > 0), the dominance variance might increase at this locus (*SI Appendix*, *SI* *Text A2*). Thus, when training the model on DH or GC lines, the weight assigned to individual SNPs can differ markedly between the two populations, explaining the fairly low prediction accuracies across populations, irrespective if model training was conducted on DH or GC lines.

Crossing with a capture line will affect linkage phases between markers and QTL and the extent of LD compared with the DH lines (Fig. 3). All GC-S_0_ plants are half-sibs and share one identical gamete. Through the subsequent selfing process, haplotypes may arise with different linkage phases and LD decay compared with those of the ancestral landrace, compromising prediction accuracies within the GC and across populations. This effect will be trait-specific and will depend strongly on the genetic makeup of the capture line. As could be seen from the experimental data, linkage phase similarities with the LS were considerably reduced in GC compared with DH lines. Prediction accuracies for plant height and especially for testcross yield were substantially reduced in the GC populations, but not for flowering time or dry matter content. We hypothesize that for the two maturity-related traits, the capture line FV2 enriched the GC populations with alleles not present in either of the two landraces at a substantial number of loci. These alleles occur with frequency 0.5 in the GC population and thus, obtain high weight in prediction compensating for the negative effects of opposing linkage phases between markers and QTL at other loci.

When predicting across landraces, accuracies were close to 0 for DH populations but >0.2 for GC lines when predicting in KE onto PE and vice versa. These results corroborate the hypothesis that prediction in the GC populations was at least partially driven by additive effects of shared FV2 haplotypes and/or their dominance over the landrace alleles (Fig. 3*C*).

### Genome-Based Improvement of Landraces.

In this study, we investigated the potential of genome-based prediction to increase the frequency of favorable alleles of target traits in landrace-derived populations. We conclude that the pure approach is to be preferred over the admixed approach, because with the admixed approach a substantial reduction in prediction accuracy must be expected unless prediction is driven by capture line alleles. When implementing the admixed approach, the choice of capture line will have a major impact on the success of the prebreeding program. It determines the mean and genetic variance of the GC population and the risk of masking favorable landrace alleles. Molecular data can inform about locus-specific allele frequencies in the ancestral landrace and the capture line, and under certain assumptions, these allele frequencies translate directly into expectations for the molecular and genetic variance in the GC population (*SI Appendix*, *SI* *Text A3*). For quantitative traits, however, many loci contribute to the genetic variance, and unless a large proportion of causal variants for the traits of interest is known, molecular parameters will provide little guidance on the choice of capture line. In this study, the phenotypic per se performance of inbred line FV2 compared with the LS and the mean of the DH lines provided a first indication for which traits the capture line might contribute alleles not present in the ancestral landrace and which type of gene action to expect. It remains to be shown for other GC populations derived from different landraces and capture lines if this pattern holds. We could also show that dominance interactions with the tester alleles differed for landrace and capture line alleles, affecting prediction accuracies in the DH and the GC populations differently. Thus, not only the capture line per se but also its interaction with the tester had a direct effect on the genetic variance accessible for selection.

In summary, the results of this study show that the pure approach has clear advantages over the admixed approach for genome-based improvement of landraces. With continuous technological advances, the application of DH technologies is likely to become routine in many plant genetic resources (19). If the production of fully inbred lines either by the DH technology or by recurrent selfing is not possible, the admixed approach is still a good alternative. The risk of masking valuable variation present in the landrace needs to be minimized by an informed choice of capture line and tester. Our study shows that the confounding effects between the alleles of the landrace and the capture line are best controlled for traits for which the capture line does not outperform the ancestral population per se or in testcrosses.

## Materials and Methods

### Plant Material.

We applied two different strategies (Fig. 1) for sampling gametes from European maize landraces. The landraces KE and PE of European flint maize were chosen of 35 landraces for this study on the basis of population-genetic analyses described by Mayer et al. (18) and phenotypic screening for variation in early-development traits assessed in field trials. DH lines were derived directly from the landraces for the first sampling strategy (pure approach) (11). For the second sampling strategy (admixed approach), we modified a scheme originally proposed by Stadler (20): pollen mixtures from the landraces were used to pollinate the capture line FV2. FV2 is an important founder line of the European flint heterotic group developed by INRA from the French landrace Lacaune and was intensively used as parent in commercial hybrids between the 1960s and 1990s. We termed this procedure “gamete capture” (GC). The GC-S_0_ plants are half-sibs, with one gamete from FV2 and the other gamete from the landrace. Subsequently, the GC-S_0_ plants were selfed to produce GC-S_1_ ears. One GC-S_1_ plant per ear was genotyped and selfed. Field evaluation was performed with the corresponding GC-S_2_ lines planted ear to row, subsequently referred to as GC-S_1:2_. For each landrace, all populations were derived from the same seed source, which we define as the ancestral landrace. Three different sets of seeds from this ancestral landrace were randomly sampled to obtain 1) the sample of the ancestral landrace (LS), 2) the landrace plants used for DH induction, and 3) the landrace plants used to pollinate the capture line. For production of testcross seed, randomly chosen lines from each population as well as FV2 and plants sampled from the ancestral landrace were hand-crossed as pollinators onto the inbred line F353 (INRA, France), a prominent line of the European dent heterotic group.

### Field Experiments and Phenotypic Data Analysis.

The DH and GC populations were evaluated in adjacent field trials connected through common checks. Field experiments for the DH populations were described in detail by Hölker et al. (11); phenotyping of the GC populations was performed analogously. Briefly, per se performance (PP) was evaluated in four environments in Germany: Roggenstein (ROG) and Einbeck (EIN) in 2017 and 2018. Two separate but adjacent sets of 8 (DH 2018) or 10 (DH and GC 2017, GC 2018) 10 × 10 lattice designs with two replicates per line were used in each environment. As common checks, we added plants sampled from the ancestral landrace (LS) as well as 15 (2017) or 4 (2018) inbred lines, including in both years the line FV2. Plots were single rows of 3 m length, with 0.75 m distance between rows, and planting density was 8.8 plants m^−2^.

Testcross performance (TP) was evaluated in two environments (ROG and EIN) in 2019. Testcrosses of DH lines were grown in four 10 × 10 lattice designs; for GC lines, a generalized *α*-lattice design with 200 entries was used. Testcrosses of the LS and of two inbred lines together with six commercial hybrids were included as checks in all trials, and FV2 was included in GC trials only. Plots were double rows of 6 m length, with 0.75 m distance between rows and planting density of 9 or 11 plants m^−2^. Sowing, fertilization, and plant protection in per se and testcross evaluation followed standard agricultural practice at the experimental stations.

The traits plant height at V6 stage (PH_V6, cm), final plant height (PH_final, cm), flowering time (FF, days from sowing until 50% of plants in the plot silked), dry matter content (DMC, percentage, only TP), and total dry matter yield (TDMY, dt/ha, only TP) at forage harvest were investigated.

We expanded the analysis described for the DH experiments in Hölker et al. (11) for joint analysis of the GC and DH experiments in a single step using the following model:

[1] $$y_{\mathrm{ijkopst}}=\mu +m_{i}+\delta_{\mathrm{Checks}}l_{j}+g_{k(ij)}+u_{o}+gu_{ko\left(ij\right)}+\delta_{DH}\{lu_{jo}+k_{p\left(o\right)}+r_{s\left(op\right)}+b_{t\left(\mathrm{ops}\right)}\}+\delta_{GC}\{lu_{\text{j}o}+k_{p\left(o\right)}+r_{s\left(op\right)}+b_{t\left(\mathrm{ops}\right)}\}+\varepsilon_{\mathrm{ijkopst}},$$

where *i* = 1, 2, 3, 4 denotes four groups (GC, DH, LS, and checks); *j* = 1, 2, 3, 4 denotes the different populations (GC_KE, GC_PE, DH_KE, and DH_PE); μ is the overall mean; $m_{i}$ is the effect of group *i*; $l_{j}$ is the effect of population *j* in groups *i* = 1 and 2; $\;\delta_{\mathrm{Checks}}$ is a dummy variable with $\delta_{\mathrm{Checks}}\;$ = 1 if the line belongs to DH or GC populations and $\delta_{\mathrm{Checks}}$ = 0 for LS or inbred lines used as checks; $\delta_{DH}$ ($\delta_{GC}$) is a dummy variable with $\delta_{DH}\;$= 1 ($\delta_{GC}\;$ = 1) if data belong to the DH (GC) experiment and $\delta_{DH}$ = 0 ($\delta_{GC}\;$= 0) otherwise; $g_{k(ij)}$ is the genotypic effect of line *k* nested in group *i* and population *j*; $u_{o}$ is the effect of environment *o*; $lu_{jo}$ is the interaction of population *j* and environment *o*; and $gu_{ko\left(ij\right)}$ is the interaction of genotype *k* and environment *o*. The effects $k_{p\left(o\right)}$, $r_{s\left(op\right)}$, $b_{t\left(\mathrm{ops}\right)}$, and $\varepsilon_{\mathrm{ijkopst}}$ refer to the effect of the lattice (nested in environments), replicate (nested in lattices in environments), incomplete block (nested in replicates in lattices in environments), and the residual error, respectively. All effects except $m_{i}$ and $l_{j}$ were treated as random. Genotype [$g_{k(ij)}$] and genotype × environment [$gu_{ko\left(ij\right)}$] variance components were modeled individually for the populations (*j* = 1, 2, 3, 4), assuming that DH and GC lines across and within landraces were stochastically independent. Residuals were assumed to be normally distributed with mean zero and four heterogeneous variances, one each for $\delta_{\mathrm{Checks}}$ = 1 and $\delta_{\mathrm{Checks}}$ = 0 in GC and DH experiments, assigning the same residual variance to all GC and DH lines within all environments. Raw data and outliers were manually curated by inspection of residual plots. The model in Eq. **1** refers to the analysis of PP. TP was analyzed analogously, adjusting for the generalized *α*-lattice design used in GC trials. Variance components and their SEs were estimated with ASReml-R package 3.0 (21). Entry-mean heritabilities were calculated for each population following Hallauer et al. (14), and SEs of heritability estimates were derived using the delta method (22). Heritabilities (*h*^2^) and variance component estimates exceeding twice their SEs were considered significant. For obtaining best linear unbiased estimates (BLUEs) of the genotypic value of each entry, the model from Eq. **1** was simplified, replacing factors $m_{i}$, $\delta_{\mathrm{Checks}}l_{j}$ with a factor separating the two experiments (DH and GC), dropping $\delta_{DH}lu_{jo}$ and $\delta_{GC}lu_{jo}$ from the model, and treating genotype as a fixed effect. This model was also used to test for significant differences (*t*-tests) between LS, DH, GC, and FV2 in linear contrasts calculated with the package asremlPlus (23). For estimating genetic covariances and genetic correlations between PP and TP for a given phenotypic trait, we expanded the model from Eq. **1** to a bivariate model treating PP as one trait and TP as the other trait. Significance of genetic covariances was tested in likelihood-ratio-tests comparing the model including the covariance with the reduced model without the covariance.

### Genetic Data Analysis.

The inbred line FV2, samples from each ancestral landrace (LS), DH lines, and GC plants were genotyped with the 600k Affymetrix Axiom Maize Array (24). The quality filtering of the SNP data for the LS and DH populations was described in detail in Hölker et al. (11) and was done analogously for the GC populations. Briefly, markers were filtered according to the best quality class (24) and an unambiguously mapped physical position in the B73 reference sequence AGPv4 (25). Markers and individuals with >10% missing values were removed. For DH lines, markers and individuals with >5% heterozygous genotype calls were removed, and the remaining heterozygous calls (0.19%) were set to missing values. For DH lines, missing values were imputed separately for each population using Beagle version 5.0 (26) with default settings. Missing values in the LS and GC were imputed, and two gametes from each individual were phased using Beagle version 5.0, with parameters iterations = 50, phase-segment = 10, and phase-states = 500. Markers were coded as counts of the FV2 allele (0: homozygous for opposite allele of FV2; 1: heterozygous; 2: homozygous for FV2 allele). In total, 1,512 genotypes (LS_KE = 48, LS_PE = 47, DH_KE = 471, DH_PE = 402, GC_KE = 274, GC_PE = 270) with 472,169 polymorphic SNPs remained for further analysis. Thereof, all DH and GC have been evaluated for PP, and a subset (DH_KE = 183, DH_PE = 173, GC_KE = 103, GC_PE = 54) has also been evaluated for TP.

### Analysis of Molecular Variance and Genetic Diversity.

We sampled 80 gametes from each population (LS, DH, and GC) and landrace with 500 replicates for comparing the number and percentage of polymorphic markers across populations.

LD was measured using *r*^2^ (27) for samples of 94 gametes within each population. We calculated *r*^2^ for pairs of SNPs within a distance of 1 Mb and used nonlinear regression to investigate the *r*^2^ decay with physical distance (28). The LD decay distance is defined as the physical distance *δ* for which the curve reaches *r*^2^ = 0.2. For estimating LD across chromosomes, we sampled 5,000 markers per chromosome with replacement for all 45 pairwise combinations of chromosomes and calculated *r*^2^ for all pairs of markers across chromosomes. Linkage phase similarities (LPS) between populations were calculated according to Schopp et al. (29). LPS according to physical distance was calculated grouping marker pairs into bins of 10 kb up to a maximum distance of 1 Mb.

Genetic distance (GD) between two genotypes was measured as GD=1−SM,  where SM is the simple matching coefficient across all SNP loci calculated as detailed by Jacobson et al. (30). We also compared allele frequencies 1) between DH and LS and 2) between the experimental and expected GC, where the expected GC was obtained by (p+1)/2, with *p* being the frequency of the FV2 allele in the respective LS. An analysis of molecular variance (31) based on Euclidean distances was used to estimate the molecular variance within and between individuals of LS, DH, and GC for each landrace. Calculations of the proportion of markers with p=0 and p≤1/3 as well as the average allele frequency for each population were based on 415,346 (KE) and 446,687 (PE) markers polymorphic across LS, DH, and GC.

### Genome-Based Prediction Model.

We performed genomic best linear unbiased prediction (GBLUP) in several scenarios for PP and TP, always applying the model

[2] y=1μ+Zu+e,

where y is a vector of BLUEs of the training set obtained from the phenotypic analysis, 1 is a vector of 1s, μ is the population mean, u is a vector of random estimated genetic values with the distribution $u\;∼\;N(0,\;U\sigma_{g}^{2})$, and Z is the corresponding incidence matrix. U is the realized relationship matrix calculated on the basis of marker data following method 1 of VanRaden (32), and $\sigma_{g}^{2}$ is the genetic variance pertaining to the GBLUP model. The matrix U was calculated considering all genotypes (both population types and landraces) as one population. The vector of residuals e is assumed to be normally distributed with a mean of zero and equal variance [$e\;∼\;N(0,\;I\sigma_{e}^{2})$], where I is the identity matrix and $\sigma_{e}^{2}$ denotes the residual variance pertaining to the GBLUP model. The relationship matrices were calculated using R [version 3.6.0 (33)] and the R-package synbreed version 0.12-9 (34). Variance components pertaining to the GBLUP model were estimated using the R-package ASReml-R version 3.0 (21).

Genomic prediction accuracy (*ρ*) is reported as the correlation between predicted and unobservable true genetic values. Estimates of *ρ* were obtained from the Pearson correlation between the observed phenotypes and the estimated genetic values divided by the square root of *h*^2^ of the prediction set (35).

### Scenarios for Genomic Prediction.

We studied the influence of the number of markers *M* and sample size *N* on *ρ* within populations by randomly sampling *M* markers using all genotypes from the respective population or sampling *N* lines without replacement from the population using all markers and carrying out 10 times fivefold cross-validation. The number of markers *M* was increased from 1,000 to 250,000. Sample size *N* was increased from 50 lines to the maximum possible number for the respective population in increments of 50. Sampling was repeated 100 times for each *M* and *N*. Prediction accuracy *ρ* was averaged across replications. The 95% quantile of *ρ* was calculated from the sampling replications. With small sample sizes *N*, *ρ* was set to “missing value” if the mixed model algorithm for a particular training set did not converge.

For comparing the prediction accuracy *ρ* within and between the DH and GC populations from the same landrace, *N* = 200 (PP) or *N* = 75 (TP) lines were sampled randomly from one population (either DH or GC) for training the model. The prediction set always comprised a disjoint set of *N* = 50 (PP) or *N* = 25 (TP) lines either from the same or from a different population. Sampling was repeated 100 times. The same sampling procedure was applied for investigating across landrace predictions using the same type of population of the other landrace as the prediction set.

## Supplementary Material

Supplementary File

## Data Availability

Seeds from all genotypes used in the study are available through material transfer agreements. The genotypic data of the inbred line FV2, 873 DH lines, 544 GC lines, and 95 landrace plants and all corresponding phenotypic data of PP and TP have been deposited in Figshare (https://doi.org/10.6084/m9.figshare.17014421) (36).
