# Supplementary material for: Theoretical and experimental assessment of genome-based prediction in landraces of allogamous crops
Source: Proc Natl Acad Sci U S A. 2022 Apr 29;119(18):e2121797119. doi: 10.1073/pnas.2121797119 (PMC9170147; doi:10.1073/pnas.2121797119)
Supplement: Supplementary File [file pnas.2121797119.sapp.pdf]

Supplementary Information for

Theoretical and experimental assessment of genome-based prediction in landraces of allogamous crops

Armin C. Hölker[a,b,1], Manfred Mayer[a,1], Thomas Presterl[b], Eva Bauer[c], Milena Ouzunova[b], Albrecht E. Melchinger[a,d,*], Chris-Carolin Schön[a,*]

[a] Plant Breeding, TUM School of Life Sciences, Technical University of Munich, 85354 Freising, Germany

[b] KWS SAAT SE & Co. KGaA, 37574 Einbeck, Germany

[c] Campus Office, TUM School of Life Sciences, Technical University of Munich, 85354 Freising, Germany

[d] Institute of Plant Breeding, Seed Science and Population Genetics, University of Hohenheim, 70593 Stuttgart, Germany

* Corresponding authors:

Chris-Carolin Schön, chris.schoen@tum.de

Albrecht E. Melchinger, melchinger@uni-hohenheim.de

[1] Armin C. Hölker and Manfred Mayer contributed equally

This PDF file includes:

Supplementary text (with Tables A1 to A3 and Figure A1)
Figures S1 to S6
Tables S1 to S3
SI References

Supplementary Text

Theoretical derivation

All theoretical results were derived under the assumption of Hardy-Weinberg Equilibrium in the ancestral landrace and absence of selection in the production of DH and GC lines. We consider three populations: the landrace sample (LS) representing randomly drawn individuals of the ancestral landrace, the doubled haploid (DH) lines derived from the ancestral landrace, and the gamete capture (GC) lines derived from a cross of individuals from the ancestral landrace with a fully inbred capture line (CL) and subsequent selfing. For all three populations, gametes from the ancestral landrace were sampled independently. Following Fehr (1) we define for the GC lines GC-$S_{m:n}$ as family in selfing generation n derived from a single individual in selfing generation m, with $m \geq 0$ and $n > m$. If $n=m$, $S_{m:n}$ refers to an individual in selfing generation m, and in this case we write S_m instead of $S_{m:n}$.

A1 Expected genetic distances among genotypes in three types of landrace derived populations

We consider the genetic distance (GD) calculated from biallelic SNP markers for pairs of genotypes (u, v), defined as $GD = 1 - SM$, where SM is the simple matching coefficient across all SNP loci calculated as detailed by Jacobson et al. (2). Table A1 presents frequencies and genetic distance values $GD(u, v)$ of pairwise genotype combinations as well as the mean genetic distance ($\overline{X}_{GD}$) for the three populations under study (LS, DH, GC).

Table A1. Frequencies and genetic distance values $GD(u, v)$ for different pairs of genotypes and respective means of these measures for the populations under study. At a given molecular marker, genotypes are coded as "11" if homozygous for the allele carried by the capture line, "10" if heterozygous, and "00" if homozygous for the alternative allele.

Population	Genotype u	Genotype v	Frequency	$GD(u,v)$
$u \neq v \in LS$	11	11	p^4	0
	11	10	$p^2 2p(1-p)$	0.5
	11	00	$p^2(1-p)^2$	1
	10	11	$2p(1-p)p^2$	0.5
	10	10	$2p(1-p)2p(1-p)$	0.5
	10	00	$2p(1-p)(1-p)^2$	0.5
	00	11	$(1-p)^2 p^2$	1
	00	10	$(1-p)^2 2p(1-p)$	0.5
	00	00	$(1-p)^4$	0
$\overline{X}_{GD}$				$2p(1-p)$
$u \neq v \in DH$	11	11	p^2	0
	11	00	$p(1-p)$	1
	00	11	$(1-p)p$	1
	00	00	$(1-p)^2$	0
$\overline{X}_{GD}$				$2p(1-p)$
$u = CL, v \in LS$	11	11	p^2	0
	11	10	$2p(1-p)$	0.5
	11	00	$(1-p)^2$	1
$\overline{X}_{GD}$				$(1-p)$
$u \neq v \in GC\text{-}S_1$	11	11	$(p+0.25(1-p))^2$	0
	11	10	$(p+0.25(1-p))0.5(1-p)$	0.5
	11	00	$(p+0.25(1-p))0.25(1-p)$	1
	10	11	$(0.5(1-p))(p+0.25(1-p))$	0.5
	10	10	$(0.5(1-p))(0.5(1-p))$	0.5
	10	00	$(0.5(1-p))0.25(1-p)$	0.5
	00	11	$0.25(1-p)(p+0.25(1-p))$	1
	00	10	$(0.25(1-p))\ 0.5(1-p)$	0.5
	00	00	$(0.25(1-p))\ 0.25(1-p)$	0
$\overline{X}_{GD}$				$0.5(1-p)(1+p)$

If the gametic array of the ancestral landrace is represented faithfully by the LS and DH populations, we expect $\overline{X}_{GD(LS)} = \overline{X}_{GD(DH)}$. Likewise, if it has been transferred faithfully to the GC-S_1 population, we have $\overline{X}_{GD(u \neq v \in GC-S_1)} = \frac{1}{4}\overline{X}_{GD(u \neq v \in LS)} + \frac{1}{2}\overline{X}_{GD(u = CL, v \in LS)}$.

A2 Quantitative genetic expectations of means and genetic variances for per se and testcross performance in three types of landrace derived populations

We consider a one-locus model with two alleles A_1 and A_2 in the panmictic ancestral landrace, and a third allele A_x in the capture line, which can be different from A_1 and A_2. Following Falconer and Mackay (3) we use a for the additive effect defined as half the difference between the genotypic values of homozygous genotypes in the ancestral landrace and d for the dominance effect. The genotypic value of the capture line is defined as $a + \Delta$. If the capture line carries a new allele, i.e. $A_x \neq A_1 \wedge A_x \neq A_2$, Δ can take any value. In case the capture line allele is already present in the landrace, we assume without loss of generality (w.l.g.) that $A_x = A_1$, which implies that $\Delta = 0$. For all possible genotypes in the three populations LS, DH and GC, the genotypic values and corresponding genotype frequencies are given in Table A2. For the GC population we provide generic solutions for different levels of inbreeding.

Table A2. Genotypic values for per se and testcross performance in terms of additive (a, Δ^*) and dominance (d_{12},d_{1x},d_{2x}) effects and genotype frequencies for the landrace sample (LS), the DH lines (DH) and the gamete capture lines (GC). Alleles segregating in LS are coded A_1 and A_2, with allele frequencies p and $(1-p)$, respectively; the allele of the capture line is coded A_x, which can be either a new allele or if not, we assume w.l.g. A_x=A_1.

**Genotype**	$\boldsymbol{A_2A_2}$	$\boldsymbol{A_1A_2}$	$\boldsymbol{A_1A_1}$	$\boldsymbol{A_2A_x}$	$\boldsymbol{A_1A_x}$	$\boldsymbol{A_xA_x}$
**Genotypic values**						
Per se	$-a$	d_{12}	a	$\frac{\Delta}{2} + d_{2x}$	$a + \frac{\Delta}{2} + d_{1x}$	$a + \Delta$
Testcrosses	$-a$	0	a	$\frac{\Delta}{2}$	$a + \frac{\Delta}{2}$	$a + \Delta$
**Frequencies**						
LS	$(1-p)^2$	$2p(1-p)$	p^2			
DH	$(1-p)$		p			
GC-S$_0$				$(1-p)$	p	
GCL-S$_{m:n}$	$\left(1-\left(\frac{1}{2}\right)^n\right)\frac{(1-p)}{2}$		$\left(1-\left(\frac{1}{2}\right)^n\right)\frac{p}{2}$	$\left(\frac{1}{2}\right)^n(1-p)$	$\left(\frac{1}{2}\right)^n p$	$\left(1-\left(\frac{1}{2}\right)^n\right)\frac{1}{2}$
GCL-S$_{m:n}$ family derived from GCL-S$_m$ line with genotype A_2A_x	$\left(1-\left(\frac{1}{2}\right)^{n-m}\right)\frac{1}{2}$			$\left(\frac{1}{2}\right)^{n-m}$		$\left(1-\left(\frac{1}{2}\right)^{n-m}\right)\frac{1}{2}$
GCL-S$_{m:n}$ family derived from GCL-S$_m$ line with genotype A_1A_x			$\left(1-\left(\frac{1}{2}\right)^{n-m}\right)\frac{1}{2}$		$\left(\frac{1}{2}\right)^{n-m}$	$\left(1-\left(\frac{1}{2}\right)^{n-m}\right)\frac{1}{2}$

* Δ can take any value, if $A_x \neq A_1 \wedge A_x \neq A_2$. For $A_x = A_1$: $\Delta = 0$.

While the same notation can be applied to genotypic values for per se and testcross performance, their interpretation is different. The tester can be an inbred line or hybrid from a different heterotic group, a different or even the same population. Assuming absence of dominance, the genotypic effect in testcrosses will be one half that of lines per se. In the presence of dominance the genotypic effect in testcrosses will depend on the respective allelic interactions between landrace, capture line and tester alleles at the given locus. Given genotypic values and their frequencies in Table A2, we obtain quantitative genetic expectations of means and variances in LS, DH and GC populations shown in Table 1 (in main manuscript) and visualized in Figure A1.

With purely additive gene action, the mean of the GC-$S_{1:2}$ population falls exactly between the mean of the DH population and the genotypic value of the capture line. To ensure readability, all genetic variances are formulated based on an additive model. For GC-$S_{1:2}$ lines per se there might be a small contribution of dominance effects to the total genetic variance. However, in the GC-$S_{1:2}$ only one fourth of the dominance variance needs to be accounted for and it has been shown for the traits under study that dominance variances are negligible compared to additive genetic variances in maize landraces (4). Thus, the primary variance of GC-$S_{1:2}$ lines, i.e. the variance between S_1 families as observed in this study, amounts to $\sigma^2_{g(GC-S_{1:2})} = \frac{3}{4}\sigma^2_A + \frac{1}{4}\sigma^2_{A*}$ with $\sigma^2_A = 2p(1-p)a^2$ and $\sigma^2_{A*} = 2\left((1-p)a + {}^{\Delta}/_{2}\right)^2$. If DH lines were derived from the GC-S_1 lines instead of selfing, a GC-$S_{1:\infty}$ population would be obtained, where seed of individual lines could be multiplied ad libitum and thus also the variance within families could be utilized (Figure A1 and Table 1 in main manuscript).

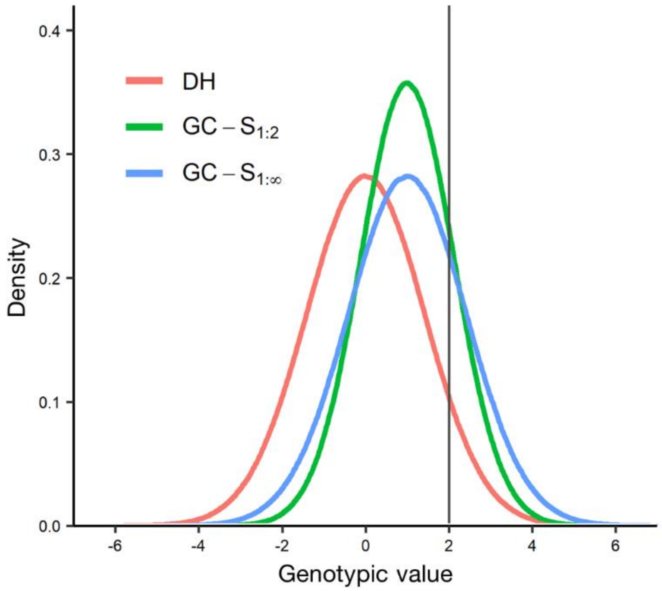

Figure A1. Stylized graphical example for the distribution of genotypic values from DH, GC-$S_{1:2}$ and GC-$S_{1:\infty}$ populations under the assumption of additive gene action and $\mu_{CL} = \mu_{DH} + 2\sigma_A$ and $\sigma_{A*} = \sqrt{2}\sigma_A$, with μ_{CL} being the performance of the capture line, indicated by the vertical line. While for GC-$S_{1:2}$ only the primary variance can be used, the total variance is available for GC-$S_{1:\infty}$ (compare Table 1 in main manuscript).

Suppose that instead of a capture line from another population a randomly sampled DH line derived from the landrace (LS) is used for gamete capture so that $A_x = A_1$ with probability p and $A_x = A_2$ with probability $1 - p$, then we obtain for the expectation $\bar{x}_{GC-S_{1:2}|A_x \in LS}$ and $\sigma^2_{A*|A_x \in LS}$:

$$E\left(\bar{x}_{GC-S_{1:2}|A_x \in LS}\right) = p\left\{pa + (1-p)\frac{d_{12}}{4}\right\} + (1-p)\left\{-(1-p)a + p\frac{d_{12}}{4}\right\} = (2p-1)a + \frac{[d]}{4}$$

$$E\left(\sigma^2_{A*|A_x \in LS}\right) = p\sigma^2_{A*|A_x = A_1} + (1-p)\sigma^2_{A*|A_x = A_2} = 2p((1-p)a + 0)^2 + 2(1-p)((1-p)a - a)^2 = 2p(1-p)a^2 = \sigma^2_A.$$

These results provide a check because the expected mean of lines derived from the LS in generation S_n, is $(2p-1)a + \left(\frac{1}{2}\right)^n [d]$ and the segregation variance within S_1 families in generation S_2 is $\frac{1}{4}\sigma^2_A$, corresponding to $\frac{1}{8}\sigma^2_A + \frac{1}{8}E\left(\sigma^2_{A*|A_x \in LS}\right)$.

A3 Comparison of molecular and additive genetic variances in DH and GC populations conditional on the frequency of the capture line allele in the ancestral landrace

The molecular variance at a given locus for the DH population is given in Table A3 as $\varsigma_{DH}^2 = 2p(1-p)$.

Table A3. AMOVA based on allele frequencies of the sample of the ancestral landrace (LS), doubled haploid (DH) and gamete capture (GC) populations. Results refer to a single biallelic locus.

Source of variation	df	Sum of squares	Expected mean squared deviations
LS	$2n_L - 1$	$2n_L(\tilde{p}_L - \tilde{p}_L^2)$	$\varsigma_{LS}^2 = 2p(1-p)$
Between genotypes	$n_L - 1$	$n_L(\tilde{p}_L + \tilde{P}_L - 2\tilde{p}_L^2)$	$\varsigma_{LS}^2[1 + F_{LS}]/2$
Within genotypes	n_L	$n_L(\tilde{p}_L - \tilde{P}_L)$	$\varsigma_{LS}^2[1 - F_{LS}]/2$
DH	$n_D - 1$	$n_D(\tilde{p}_D + \tilde{P}_D - 2\tilde{p}_D^2)$	$\varsigma_{DH}^2 = 2p(1-p)$
GC-S1	$2n_G - 1$	$2n_G(\tilde{p}_G - \tilde{p}_G^2)$	$\varsigma_{GC-S_1}^2 = (1+p)(1-p)/2$
Between genotypes	$n_G - 1$	$n_G(\tilde{p}_G + \tilde{P}_G - 2\tilde{p}_G^2)$	$\varsigma_{GC-S_1}^2[1 + F_{GC-S_1}]/2$
Within genotypes	n_G	$n_G(\tilde{p}_G - \tilde{P}_G)$	$\varsigma_{GC-S_1}^2[1 - F_{GC-S_1}]/2$

n_L, n_D, n_G number of genotypes sampled from population LS, DH and GC, respectively
$\tilde{p}_L, \tilde{p}_D, \tilde{p}_G$ observed frequency of reference allele A_1 (fixed in FV2) in the sample from LS, DH and GC, respectively
$\tilde{P}_L, \tilde{P}_D, \tilde{P}_G$ observed frequency of genotype A_1A_1 in the sample from LS, DH and GC, respectively
p frequency of A_1 in the LS and expected frequency in the DH population under Mendelian inheritance
F_{LS}, F_{GC} inbreeding coefficient of genotypes of LS and GC, respectively. For GC-S$_1$ genotypes the expectation is $F_{GC-S_1} = p/(1+p)$, because $F = \frac{H_S - H_I}{H_S}$, with H_S being the expected proportion of heterozygous genotypes under Hardy-Weinberg equilibrium and H_I being the observed proportion of heterozygous individuals, with expectations $H_S = 2p_{GC-S_1}(1 - p_{GC-S_1}) = 2\left(\frac{p+1}{2}\right)\left(1 - \frac{p+1}{2}\right) = 2\left(\frac{p+1}{2}\right)\left(\frac{1-p}{2}\right)$ and $H_I = \left(\frac{1-p}{2}\right)$ for GC-S$_1$ genotypes.

For the molecular variance between GC-S$_1$ lines, which corresponds to the primary variance of the GC-S$_{1:2}$ population, we have (Table A3):

$$\varsigma_{GC-S_1}^2 = \frac{\varsigma_{GC-S_1}^2(1+F_{GC-S_1})}{2} = \left[\frac{(1+p)(1-p)}{2}\right]\left[\frac{1+p/(1+p)}{2}\right] = \frac{(1+p)(1-p)+(1+p)(1-p)p/(1+p)}{4} = \frac{(1+p)(1-p)+(1-p)p}{4} = \frac{(1+2p)(1-p)}{4}.$$

Thus, we have $\varsigma_{GC-S1}^2 > \varsigma_{DH}^2$ if and only if

$$\frac{(1+2p)(1-p)}{4} > 2p(1-p) \iff \frac{(1+2p)}{4} > 2p \iff (1+2p) > 8p \iff 1 > 6p \iff p < \frac{1}{6}$$

The additive genetic variance of the DH population is

$$\sigma_{g(DH)}^2 = 2\sigma_A^2 = 4p(1-p)a^2.$$

For the GC-S$_{1:2}$ population only the primary variance is available. Under the assumption of $A_x = A_1$, which implies that $\Delta = 0$, we have

$$\sigma_{g(GC-S_{1:2}\,primary\,variance)}^2 = \frac{3}{4}\sigma_A^2 + \frac{1}{4}\sigma_{A^*}^2 = \frac{3}{4}[2p(1-p)a^2] + \frac{1}{4}\left[2\big((1-p)a\big)^2\right] \text{ or}$$

$$\sigma_{g(GC-S_{1:2}\,primary\,variance)}^2 = \frac{1}{2}(1-p)(3p+1-p)a^2 = \frac{1}{2}(1-p)(1-2p)a^2.$$

Hence,

$\sigma^2_{g(GC-S_{1:2}\ primary\ variance)} > \sigma^2_{g(DH)}$ if and only if

$\frac{1}{2}(1-p)(1-2p)a^2 > 4p(1-p)a^2 \iff (1-2p) > 8p \leftrightarrow p < \frac{1}{6}$.

Supplemental Figures and Tables

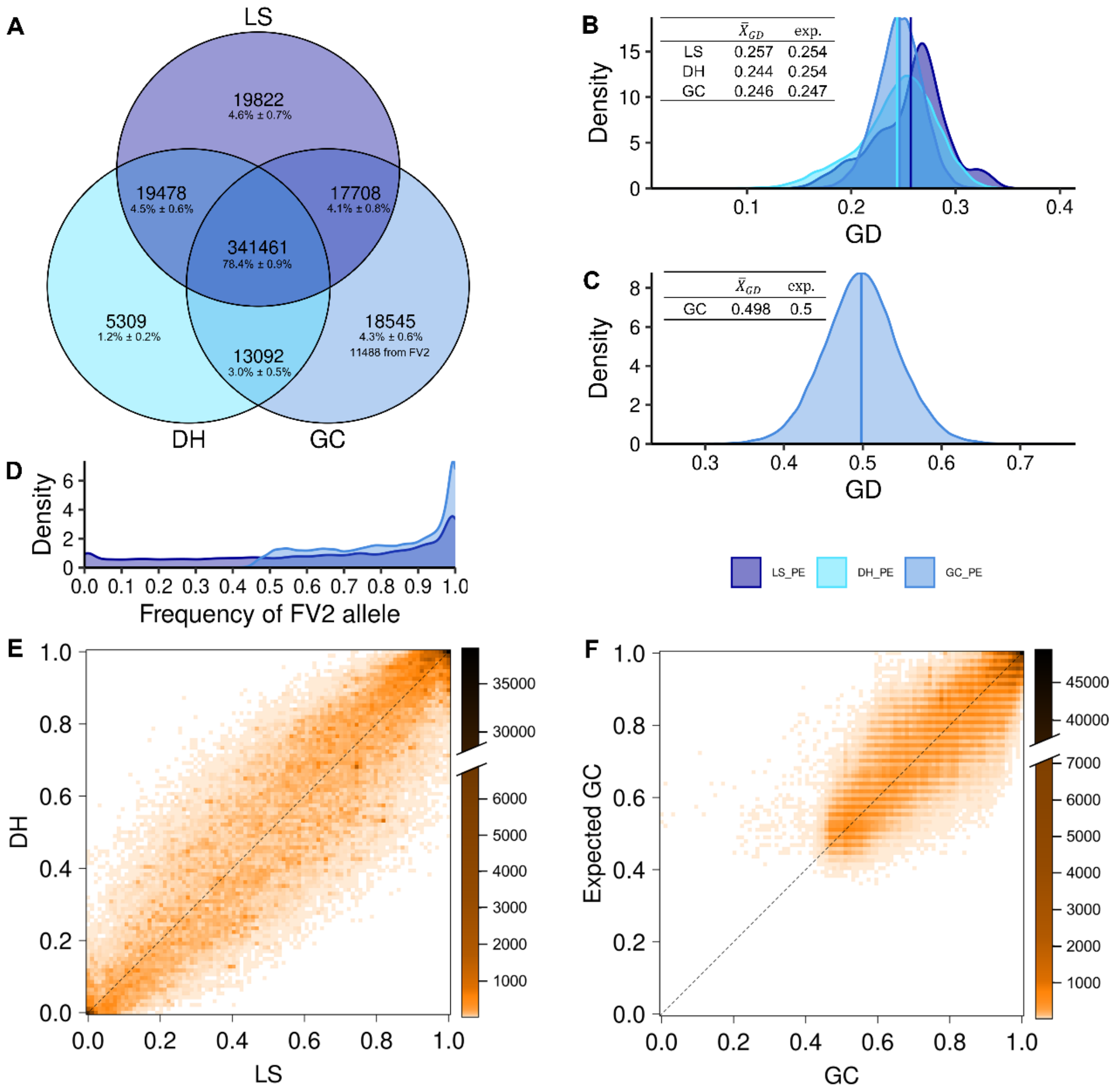

Fig. S1. Venn diagram of number and percentage of marker polymorphisms shared by and exclusive to the sample of the ancestral landrace (LS), doubled haploid (DH) lines, and gamete capture (GC) lines of landrace Petkuser Ferdinand Rot (PE, A); means and estimated densities of genetic distances (GD) between genotypes within LS, DH, and GC using all markers (B) and between GC lines and FV2 using only markers for which DH and LS were monomorphic for the allele not carried by FV2 (C); estimated density of the frequency of the FV2 allele in LS and GC (D); allele frequencies in DH vs. LS (E) and expected frequencies in GC (calculated from LS and known FV2 genotype) vs. observed GC (F). The calculated numbers of marker polymorphisms (A) are the result of sampling 80 gametes per population with 500 replications and are shown as the absolute number and percentage of polymorphic markers (± standard deviation). In GC the number of polymorphic markers resulting from the cross with FV2 (LS and DH monomorphic for the allele not carried by FV2) is shown as the average across 500 sampling replications. The tables in B and C show the means of the genetic distances and their expected values (calculated from LS allele frequencies). Figures B – F are based on the whole set of lines, i.e. N = 47 (LS), N = 402 (DH) and N = 270 (GC).

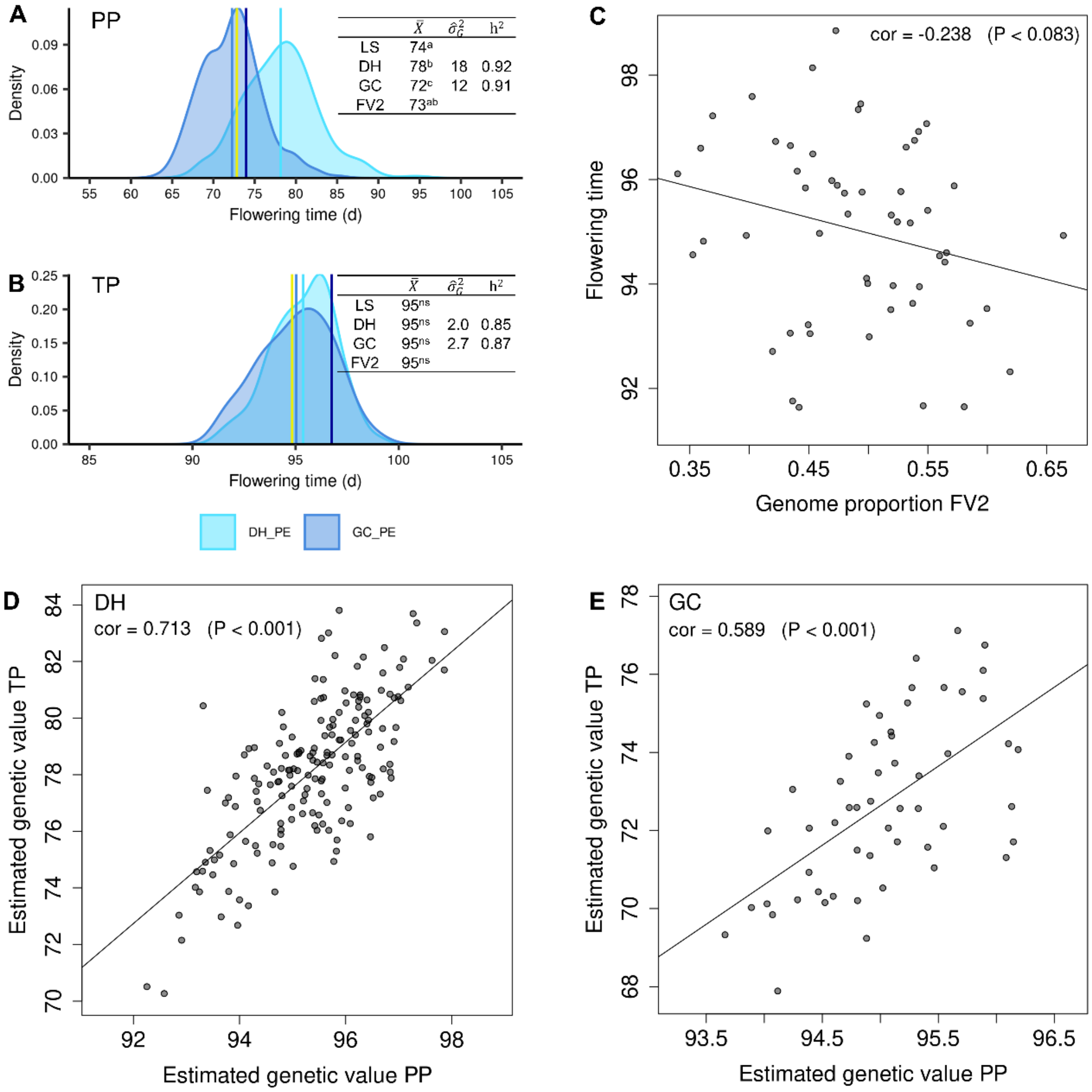

Fig. S2. Estimated densities showing the distribution of phenotypic values for per se performance (PP, A) and testcross performance (TP, B) of the doubled-haploid (DH) and gamete capture (GC) lines for landrace Petkuser Ferdinand Rot (PE), scatterplots of proportion of FV2 genome vs. TP for flowering time (C), estimated genetic values of PP vs. estimated genetic values of TP for flowering time in DH (D) and GC (E) lines. In A and B, the means (vertical lines) of the landrace sample (LS, dark green) and the capture line FV2 (yellow) are indicated and the table shows the means ($\bar{X}$), genetic variances ($\hat{\sigma}_g^2$), and heritabilities (h^2). Means with a shared letter are not significantly different (P > 0.05). Panels C, D, and E indicate the Pearson correlation coefficients and corresponding P-values of the shown correlations.

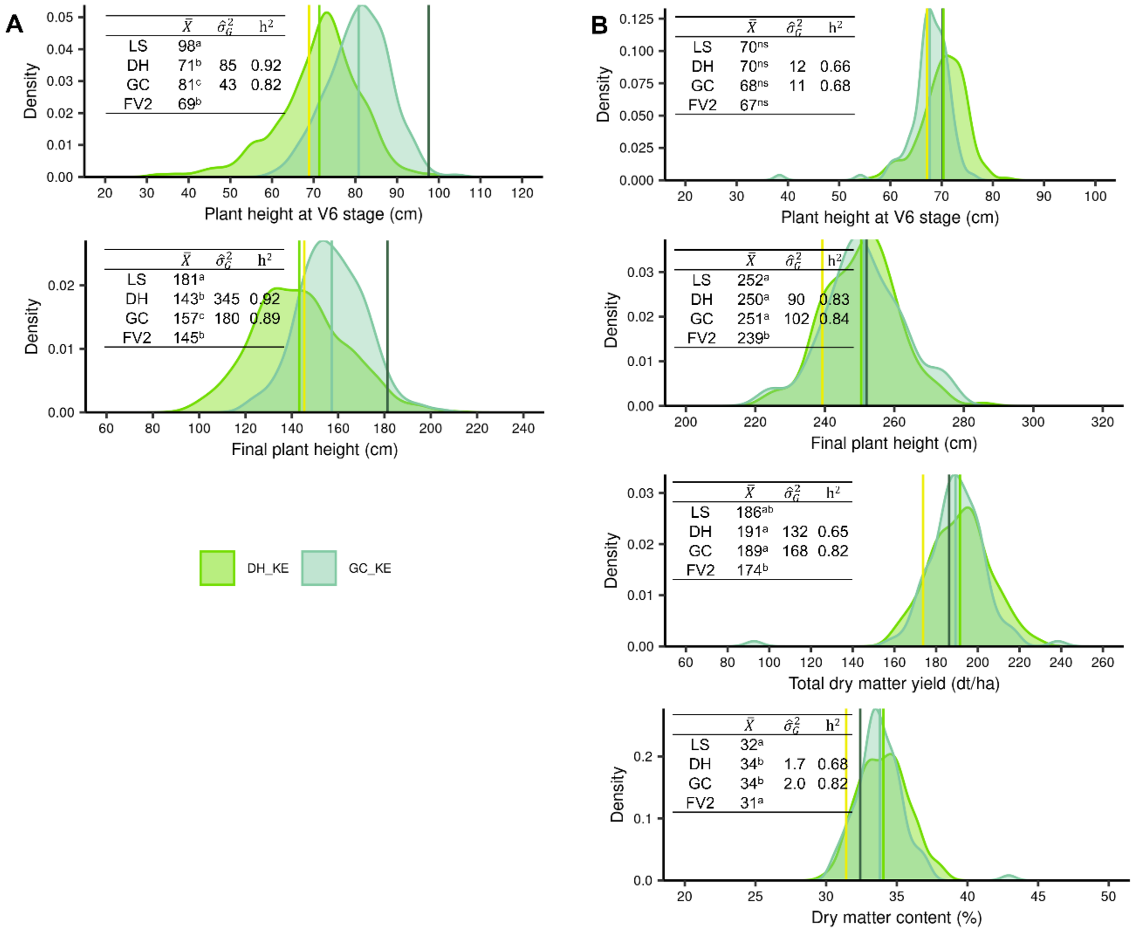

Fig. S3. Estimated densities showing the distribution of phenotypic values for per se performance (PP, A) and testcross performance (TP, B) of the doubled-haploid (DH) and gamete capture (GC) lines for landrace Kemater Landmais Gelb (KE). Means are indicated as vertical lines for the landrace sample (LS, dark green) and the capture line FV2 (yellow) for traits plant height at V6 stage and final plant height in PP and TP and dry matter content and total dry matter yield in TP. The table in each graph (A, B) gives the means ($\bar{X}$), genetic variances ($\hat{\sigma}_g^2$), and heritabilities (h^2) for the respective trait. Means with a shared letter are not significantly different (P > 0.05).

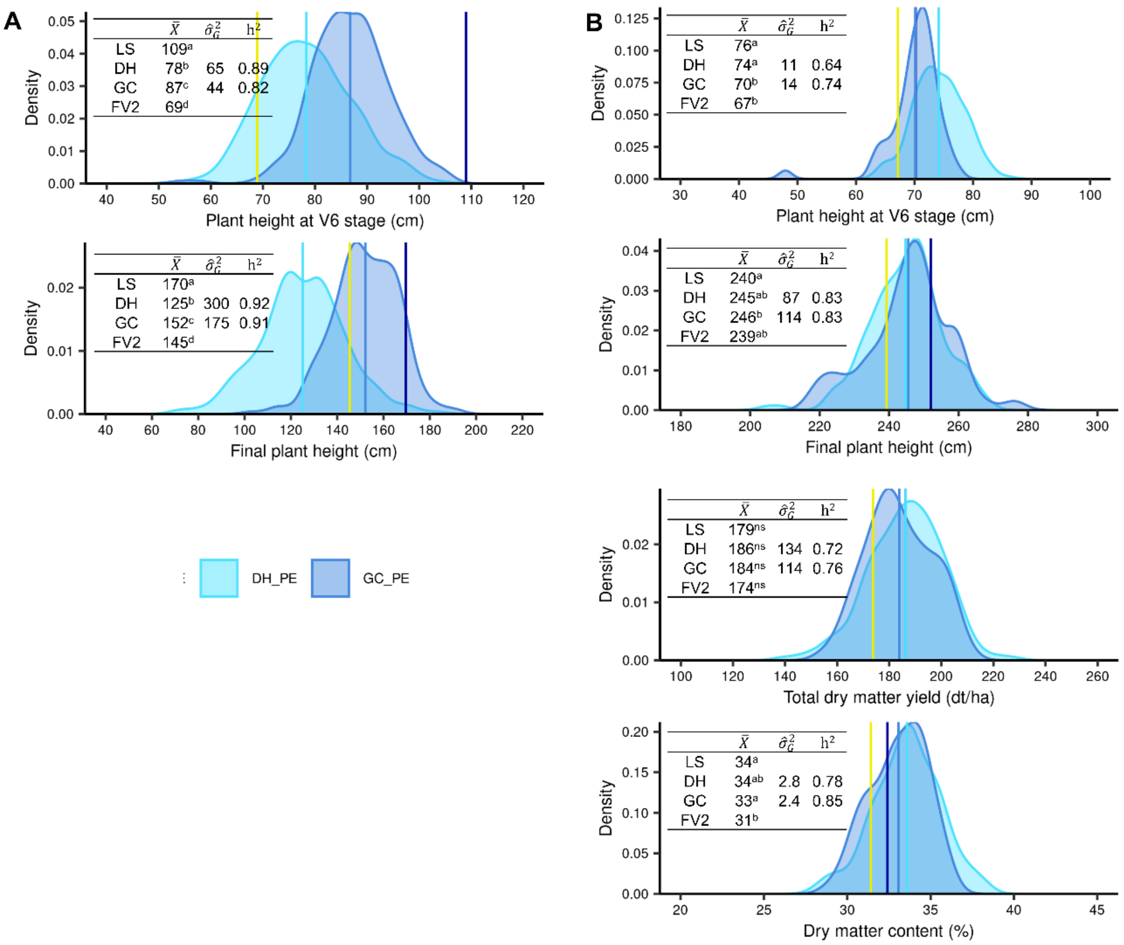

Fig. S4. Estimated densities showing the distribution of phenotypic values for per se performance (PP, A) and testcross performance (TP, B) of the doubled-haploid (DH) and gamete capture (GC) lines for landrace Petkuser Ferdinand Rot (PE). Means are indicated as vertical lines for the landrace sample (LS, dark blue) and the capture line FV2 (yellow) for traits plant height at V6 stage and final plant height in PP and TP and dry matter content and total dry matter yield in TP. The table in each graph (A, B) gives the means ($\bar{X}$), genetic variances ($\hat{\sigma}_g^2$), and heritabilities (h^2) for the respective trait. Means with a shared letter are not significantly different (P > 0.05).

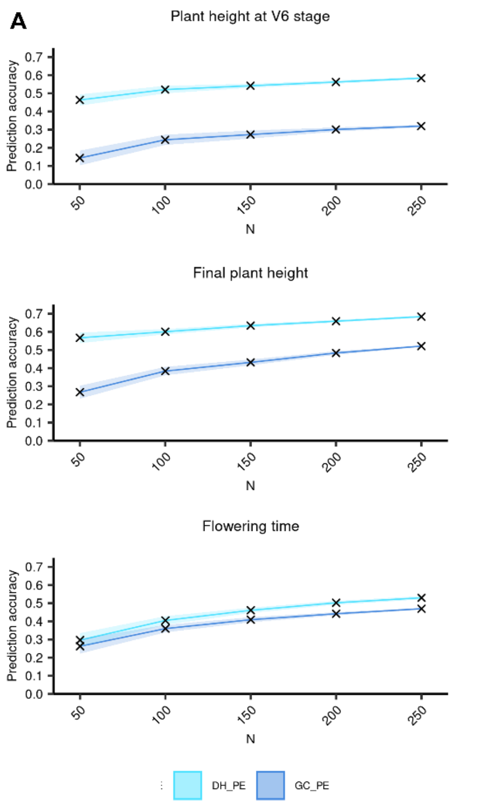

B

		DH_PP	GC_PP	DH_TC
N_{max}		402	270	173
	PH_V6	0.61	0.36	0.57
	PH_final	0.72	0.54	0.69
ρ	FF	0.60	0.48	0.67
	DMC	-	-	0.52
	TDMY	-	-	0.58

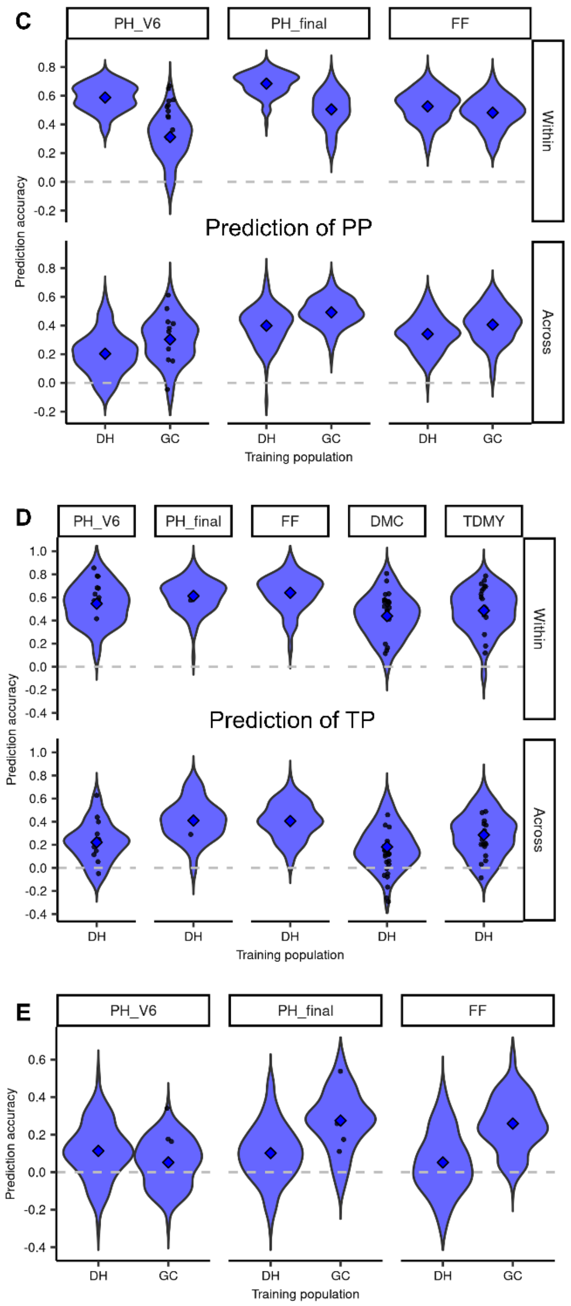

Fig. S5. Prediction accuracy (*ρ*) in landrace Petkuser Ferdinand Rot (PE) for per se performance (PP) in the doubled haploid (DH) and gamete capture (GC) lines as a function of sample size *N* (A); for prediction of PP and testcross performance (TP, only DH) at the maximum available number of lines (N_{max}) (B); for predictions within and across populations for PP (C) and TP (D), and for across landrace prediction for PP from PE (training on Kemater Landmais Gelb, KE) (E). Traits are plant height at V6 stage (PH_V6), final plant height (PH_final), and flowering time (FF) in PP and TP and dry matter content (DMC) and total dry matter yield (TDMY) in TP. For each *N* (A), sampling of lines was repeated 100 times and 10-times 5-fold cross-validation was carried out within each sample, yielding the basis for calculating the presented means and 95% quantiles (shaded area around the curve). Prediction across and within populations as well as across landraces was carried out by randomly sampling N = 200 and N = 75 lines for training in PP (C, E), and TP (D), respectively, for predicting N = 50 (PP, C, E), or N = 25 (D) genotypes of the same or corresponding population (C, D), or the same population of the other landrace (E). Sampling was repeated 100 times. The violin plots (C, D, E) show all 100 values, with the diamond indicating the mean. Black dots show values of the prediction accuracy estimated from models where the genomic variance estimate was not significant (likelihood-ratio-test, P > 0.05).

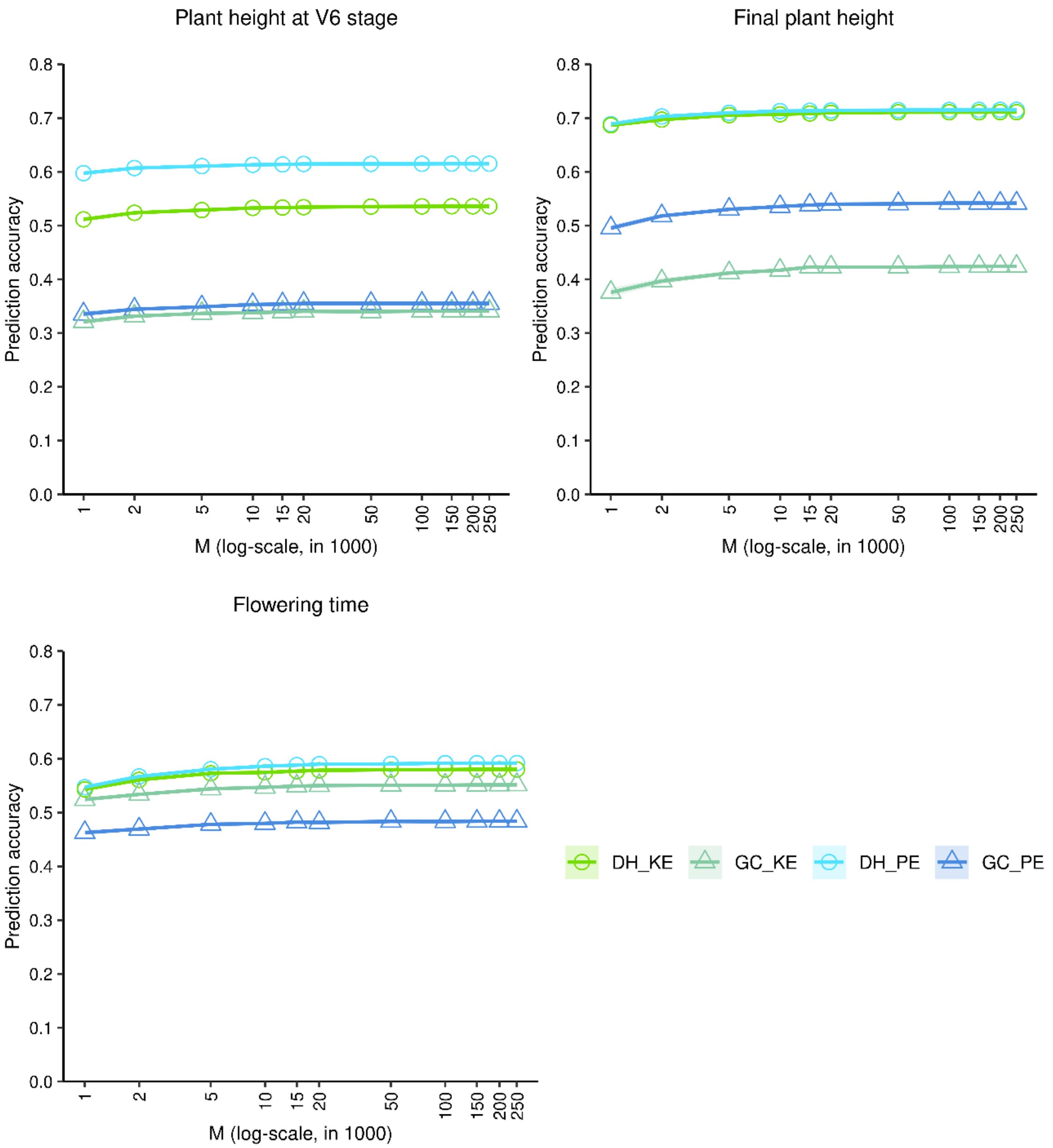

Fig. S6. Prediction accuracy (*ρ*) in landraces Kemater Landmais Gelb (KE) and Petkuser Ferdinand Rot (PE) for per se performance (PP) in the doubled haploid (DH) and gamete capture (GC) lines vs. number of markers (*M*) used for calculating the relationship matrix in the mixed model for traits plant height at V6 stage, final plant height and flowering time. For each *M*, sampling was repeated 100 times and for each sample, 5-fold cross-validation was repeated 10 times, yielding the basis for calculating the means and 95% quantiles for *ρ*, shown as shaded area around the curves.

Table S1. Analysis of molecular variance (AMOVA) based on Euclidean distances between gametes from the ancestral landrace sample (LS), doubled-haploid (DH) and gamete capture (GC) lines from landraces Kemater Landmais Gelb (KE) and Petkuser Ferdinand Rot (PE) with estimates of the molecular variance. All mean squared deviations (MSD) were significant (10,000 permutations, P < 0.05).

		**KE**			**PE**		
	Source of variation	df	MSD	Variance	df	MSD	Variance
LS							
	Between genotypes	47	53621	56457	46	56347	60672
	Within genotypes	48	59293		47	64997	
DH		470	123741	61871	401	115216	57608
GC-S_1							
	Between genotypes	273	68072	56283	269	70771	58137
	Within genotypes	274	44495		270	45502	

Table S2. Phenotypic Pearson correlation (r_p) and genetic correlation (r_g) between per se performance (PP) and testcross performance (TP), mean of prediction accuracy for PP (ρ_{PP}), as well as accuracy for prediction of TP when training on PP for plant height at V6 stage, final plant height, and flowering time in doubled-haploid (DH) and gamete capture (GC) populations derived from landraces Kemater Landmais Gelb (KE) and Petkuser Ferdinand Rot (PE). All correlation coefficients are significant ($P < 0.05$), unless indicated by “ns”.

	r_p	r_g	ρ_{PP}	$\rho_{TP(PP)}$
		**Plant height at V6 stage**		
DH_KE	0.49	0.65	0.53	0.35
GC_KE	ns	ns	0.41	0.10
DH_PE	0.57	0.79	0.59	0.55
GC_PE	0.36	0.45	0.37	0.19
		**Final plant height**		
DH_KE	0.63	0.76	0.62	0.46
GC_KE	0.63	0.78	0.52	0.33
DH_PE	0.49	0.66	0.60	0.46
GC_PE	0.58	0.70	0.57	0.46
		**Flowering time**		
DH_KE	0.62	0.84	0.48	0.41
GC_KE	0.56	0.69	0.49	0.32
DH_PE	0.63	0.84	0.55	0.61
GC_PE	0.64	0.66	0.37	0.22

Table S3. Prediction accuracy (ρ) for per se performance using different training sets comprising either a fixed number of lines from the same population (e.g. DH, ρ_{one}) or using a fixed number of lines from the same population and adding all lines from the second population (e.g. GC, ρ_{both}). Numbers of lines was either N = 350 (DH) or N = 200 (GC) for ρ_{one} or between 602 and 671 for ρ_{both}. Prediction was carried out for landraces Kemater Landmais Gelb (KE) and Petkuser Ferdinand Rot) and sampling was repeated 100 times. Traits are plant height at V6 stage, final plant height and flowering time.

	N_{one}	N_{both}	ρ_{one}	ρ_{both}
**Plant height at V6 stage**				
DH_KE	350	624	0.55	0.57
GC_KE	200	671	0.35	0.42
DH_PE	350	620	0.63	0.64
GC_PE	200	602	0.31	0.34
**Final plant height**				
DH_KE	350	624	0.70	0.72
GC_KE	200	671	0.39	0.43
DH_PE	350	620	0.73	0.73
GC_PE	200	602	0.50	0.47
**Flowering time**				
DH_KE	350	624	0.58	0.61
GC_KE	200	671	0.55	0.51
DH_PE	350	620	0.60	0.62
GC_PE	200	602	0.48	0.56

SI References

1. W. Fehr, “Genetic Principles” in *Principles of Cultivar Development: Theory and Technique*, (Macmillian Publishing Company, 1991), pp. 31–33.

2. A. Jacobson, L. Lian, S. Zhong, R. Bernardo, Minimal Loss of Genetic Diversity after Genomewide Selection within Biparental Maize Populations. *Crop Science* **55**, 783–789 (2015).

3. D. Falconer, T. Mackay, *Introduction to quantitative genetics*, 4th Ed. (Longmann Scientific & Technical, 1996).

4. C. J. Yang, *et al.*, The genetic architecture of teosinte catalyzed and constrained maize domestication. *PNAS* **116**, 5643–5652 (2019).
